# Hsp90 Interacts Specifically with Viral RNA and Differentially Regulates Replication Initiation of *Bamboo mosaic virus* and Associated Satellite RNA

**DOI:** 10.1371/journal.ppat.1002726

**Published:** 2012-05-24

**Authors:** Ying Wen Huang, Chung Chi Hu, Ming Ru Liou, Ban Yang Chang, Ching Hsiu Tsai, Menghsiao Meng, Na Sheng Lin, Yau Heiu Hsu

**Affiliations:** 1 Graduate Institute of Biotechnology, National Chung Hsing University, Taichung, Taiwan; 2 Institute of Plant and Microbial Biology, Academia Sinica, Taipei, Taiwan; 3 Institute of Biochemistry, National Chung Hsing University, Taichung, Taiwan; University of Kentucky, United States of America

## Abstract

Host factors play crucial roles in the replication of plus-strand RNA viruses. In this report, a heat shock protein 90 homologue of *Nicotiana benthamiana*, NbHsp90, was identified in association with partially purified replicase complexes from BaMV-infected tissue, and shown to specifically interact with the 3′ untranslated region (3′ UTR) of BaMV genomic RNA, but not with the 3′ UTR of BaMV-associated satellite RNA (satBaMV RNA) or that of genomic RNA of other viruses, such as *Potato virus X* (PVX) or *Cucumber mosaic virus* (CMV). Mutational analyses revealed that the interaction occurs between the middle domain of NbHsp90 and domain E of the BaMV 3′ UTR. The knockdown or inhibition of NbHsp90 suppressed BaMV infectivity, but not that of satBaMV RNA, PVX, or CMV in *N. benthamiana*. Time-course analysis further revealed that the inhibitory effect of 17-AAG is significant only during the immediate early stages of BaMV replication. Moreover, yeast two-hybrid and GST pull-down assays demonstrated the existence of an interaction between NbHsp90 and the BaMV RNA-dependent RNA polymerase. These results reveal a novel role for NbHsp90 in the selective enhancement of BaMV replication, most likely through direct interaction with the 3′ UTR of BaMV RNA during the initiation of BaMV RNA replication.

## Author Summary

Heat shock protein 90 (Hsp90) is a highly conserved molecular chaperone in prokaryotes and eukaryotes, and regulates diverse cellular processes through ensuring the correct folding of numerous client proteins. However, there are no reports of direct interactions between Hsp90 with viral RNA. Here, we report that a new member of the Hsp90 proteins of *Nicotiana benthamiana*, NbHsp90, specifically interacts with the 3′ UTR of *Bamboo mosaic virus* (BaMV) genomic RNA, but not with the 3′ UTR of BaMV-associated satellite RNA or that of other viruses. We further demonstrate that NbHsp90 specifically involves in the immediately early stage of BaMV RNA replication. NbHsp90 directly interacts with the BaMV 3′ UTR through the domain E, the key structural differences that distinguishes the BaMV 3′ UTR from the satBaMV 3′ UTR, which might contribute to NbHsp90's differential requirement for BaMV and satBaMV replication. Our work revealed a new role of Hsp90 in the interaction with RNA molecules, and demonstrated the differential requirement of Hsp90 in the replication of BaMV and satBaMV RNAs, which provide additional leverage for understanding the complex interactions between host, virus and its associated satellite RNA.

## Introduction

Viruses have limited coding capacity and require a multitude of host factors to support their biological functions during the infection cycle [1]–[4]. Researchers have used various experimental approaches in their search for host factors specifically required for viral replication. These approaches have included genome-wild screening of host factors that affect viral replication using yeast mutants [5]–[8], the direct identification of host proteins co-purified with viral replicase complexes [9]–[11], and the capturing of host proteins that bind specifically to viral RNA [12], [13]. The 3′-untranslated regions (3′ UTRs) of viral genomic RNAs contain *cis*-acting sequences required for the initiation of minus-strand RNA synthesis during replication [14]–[16], and hence 3′ UTRs have been used in several studies as a bait to identify host factors involved in viral replication processes. A number of host factors have been shown to physically interact with the *cis*-acting elements of viral RNA from *Brome mosaic virus*, *Hepatitis C virus* (HCV), tombusvirus and *Tobacco mosaic virus* (TMV), and are required for the efficient replication of these viruses [13], [17]–[20]. Efforts toward identifying and characterizing the various host factors required for viral RNA replication will help shed light on the molecular biology of viruses, and thus provide a valuable resource for the development of antiviral strategies.

*Bamboo mosaic virus* (BaMV), a member of the genus *Potexvirus*, has a single-stranded, positive-sense RNA genome of approximately 6,400 nt [excluding the 3′ poly(A) tail] with a 5′ cap-structure and a 3′ poly(A) tail. The five open reading frames (ORFs 1 to 5) of the BaMV genome encode ORF1 protein (155 kDa), TGBp1 (28 kDa), TGBp2 (13 kDa), TGBp3 (6 kDa), and the capsid protein (25 kDa), respectively [21], [22]. The BaMV ORF1 protein consists of three functional domains: the capping enzyme domain [23], [24], the helicase-like domain [25], and the RNA-dependent RNA polymerase (RdRp) core domain [26]. The 3′ UTR from BaMV genomic RNA can fold into four independent stem-loops (domains A to D) and a tertiary pseudoknot structure (domain E), both of which are important for BaMV replication [27]. In addition to genomic and subgenomic RNAs, the satellite RNA associated with BaMV (satBaMV RNA) is dependent on BaMV for its replication, encapsidation, and systemic movement [28]. The satBaMV RNA is 836 nt in length [excluding the 3′ poly(A) tail] and has an ORF for a 20-kDa protein [28]. The satBaMV RNA 3′ UTR structure comprises three stem-loops, SLA, SLB, and SLC, and two *cis*-acting elements responsible for efficient replication [29]. Although the satBaMV 3′ UTR sequence and structural elements mimic those of the BaMV 3′ UTR for recognition by the BaMV RdRp complexes, discrepancies between these two 3′ UTRs are significant, most notably the lack of a pseudoknot structure in the satBaMV RNA 3′ UTR [29], [30]. Thus, whether satBaMV RNA shares the same set of replicase complexes with BaMV or uses another set of replicase complexes (possibly with different host factors) for replication remains an open question.

Heat shock protein 90 (Hsp90) is a highly conserved molecular chaperone in prokaryotes and eukaryotes, and regulates diverse cellular processes through ensuring the correct folding of numerous client proteins [31], [32]. Hsp90 client proteins are involved in signal transduction, steroid signaling, protein trafficking, and stress response [32]–[34]. There is increasing evidence that many viruses recruit the Hsp90 chaperone function for assistance with viral protein synthesis, maturation, and stabilization [35]. For example, Hsp90 is essential for *Flock house virus* RNA polymerase synthesis and for efficient infection in *Drosophila* cells [36], [37]. Hsp90 is also essential for maturation of HCV non-structural protein NS2/3, stability of NS3, and the assembly of replicase complexes [38], [39]. Hsp90 plays a role in nuclear import and assembly of the *Influenza A virus* polymerase complex by binding to PB1 and PB2 polymerase subunits [40], [41]. Activated Hsp90 binds to hepatitis B virus core protein and facilitates capsid formation [42]. The dengue virus receptor-complex, comprising Hsp90 and Hsp70, is important for virus entry [43]. Another molecular chaperone family, the Hsp70 proteins, plays a key role in replication of plant viruses such as tombusvirus [11], [44], [45]. To date, however, there has been no evidence of a direct interaction between Hsp90 and viral RNA or for involvement of such an interaction in viral RNA replication.

In this article, we report a direct and specific interaction between Hsp90 and the 3′ UTR of BaMV RNA, and illustrate a novel and required function for Hsp90 during the early stages of BaMV replication by using virus-induced gene silencing and Hsp90-specific inhibitors. Our findings reveal a unique and specific role for Hsp90 in viral RNA recognition and replication, and suggest that replicase complexes recruit different host factors for replication of BaMV and satBaMV. We propose a model to illustrate the stages at which Hsp90 is most likely involved in BaMV RNA replication, and to further describe the differential requirement of Hsp90 in the replication of BaMV and satBaMV RNAs.

## Results

### Specific interaction of NbHsp90 with BaMV 3′ UTR

**Figure 1:**
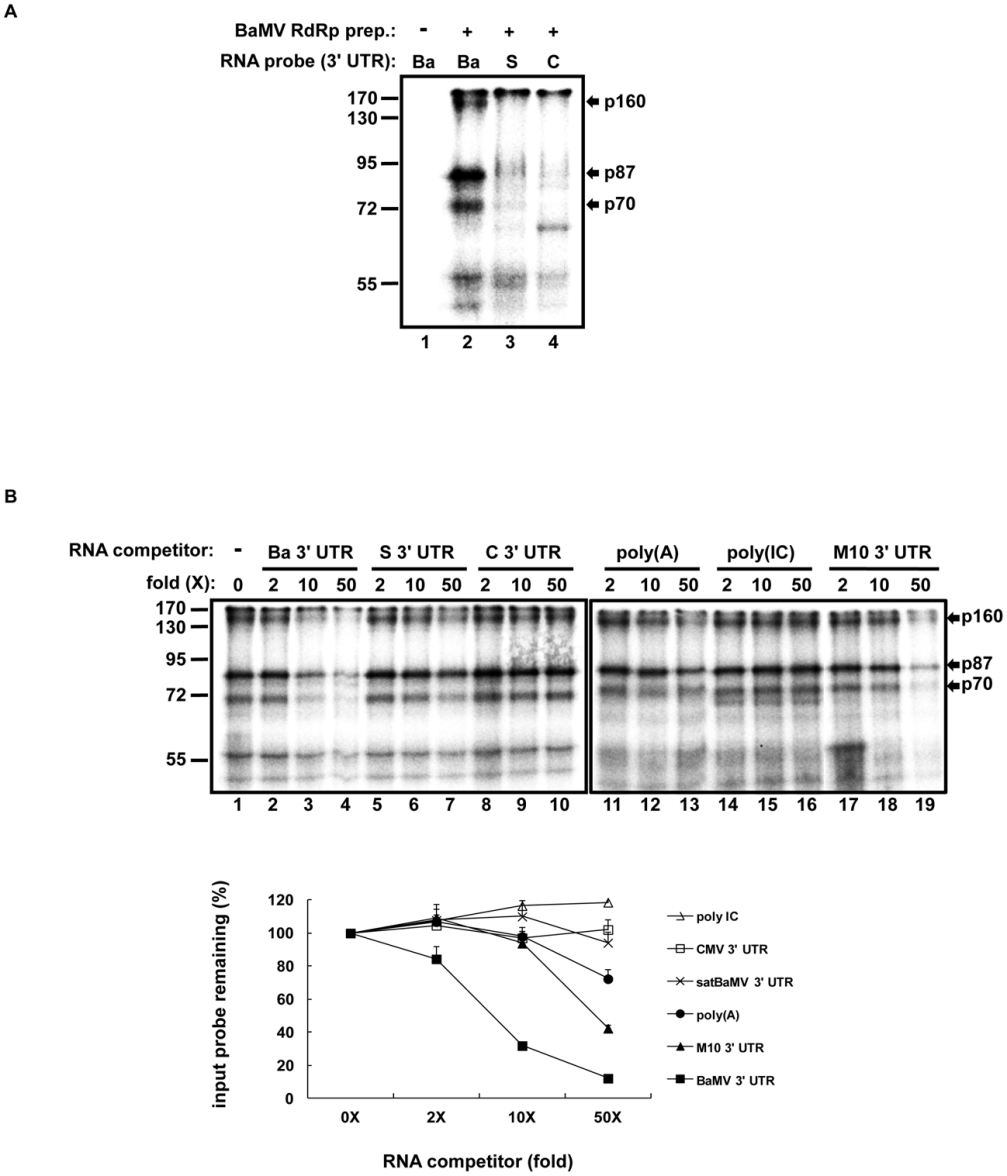
Identification of host factors that specifically interact with the BaMV 3′ UTR in BaMV RdRp preparation. (**A**) Detection of host factors interacting with the 3′ UTR of BaMV, satBaMV, or CMV by UV cross-linking assay. The BaMV (Ba), satBaMV (S), and CMV (C) 3′ UTR probes were labeled with [α-^32^P] UTP and individually incubated with the membrane fraction containing BaMV RdRp activity (BaMV RdRp prep.) purified from BaMV-infected *N. benthamiana* (lanes 2–4). The RNA-bound proteins were separated by electrophoresis on 10% SDS-polyacrylamide gels (PAG). In this experiment, the unbound RNA were digested away by RNase A and T1, leaving only a few ^32^P-labeled nucleotides covalently linked to target proteins, which might slightly increase the molecular weight in the range of several kDa. Thus the relative molecular weights of the proteins were estimated by the mobility in the gel in comparison with pre-stained molecular weight markers, whose sizes (kDa) are indicated at the left of the panel. Arrows indicate the position of the candidate proteins, p160, p87, and p70, that interact with the BaMV 3′ UTR in preference to the satBaMV and CMV 3′ UTRs. (**B**) Characterization of binding specificity between the BaMV 3′ UTR and candidate proteins by competitive UV cross-linking assay. BaMV RdRp prep. was incubated with the BaMV 3′ UTR probe in the presence of 2-, 10-, and 50-fold molar excesses of unlabeled BaMV 3′ UTR competitor (lanes 2–4), the satBaMV 3′ UTR (lanes 5–7), the CMV 3′ UTR (lanes 8–10), and the M10 3′ UTR (lanes 17–19) and three, 15, and 75 ng of poly(A) (lanes 11–13) or poly(IC) (lanes 14–16) competitors. Bindings without competitors are indicated as “−” (lane 1). A quantification of band intensities corresponding to p87 is shown in a plot below. The band-intensity for treatment without competitor RNA is defined as 100%. All assays were performed in at least three independent experiments.

Results from previous studies suggested that satBaMV evolved a distinct 3′ UTR RNA structure compared to that of BaMV [29]. BaMV 3′ UTR comprises four stem-loops and one pseudoknot, while satBaMV 3′ UTR comprises three stem-loops (Figures S1A and S1B) [27], [29]. This distinction would suggest more efficient replication and the requirement of different host factors for replication. To test these hypotheses, we performed UV cross-linking assays to examine the binding of components in BaMV RdRp preparations with BaMV, satBaMV, and CMV 3′ UTRs (Figure 1A). The CMV 3′ UTR, containing a tRNA-like structure absent from both the BaMV and satBaMV 3′ UTR, was used as a control RNA for checking binding specificity (Figure 1A, lane 4). Figure 1A shows differential binding of proteins in BaMV RdRp preparations with BaMV and satBaMV 3′ UTRs. Three proteins with estimated relative molecular weights of about 70, 87, and 160 kDa (termed p70, p87, and p160, respectively) were found to have high affinity for the BaMV 3′ UTR (Figure 1A, lane 2, indicated by arrows). To test the specificity of the interaction between these proteins and the BaMV 3′ UTR, UV cross-linking assays were performed in the presence of various unlabeled competitor RNAs (Figure 1B). The intensities of p87 bound with the BaMV 3′ UTR were quantified and the percentages of the binding signals relative to that in the absence of competitor RNA (Figure 1B, lane 1) were plotted. The unlabeled BaMV 3′ UTR was an effective competitor; it was able to compete out about 70% of the labeled BaMV 3′ UTR probe at 10-fold molar excess (Figure 1B, lane 3). By contrast, the satBaMV 3′ UTR, the CMV 3′ UTR, and poly(IC) were unable to compete with the BaMV 3′ UTR probe efficiently, even at 50-fold molar excess (Figure 1B, lanes 7, 10 and 16). These results indicated that p70, p87, and p160 specifically interact with the BaMV 3′ UTR, but not with the 3′ UTRs of satBaMV and CMV, and not with double-stranded RNA. One of the major differences between the BaMV and satBaMV RNA 3′ UTR structures is the presence of a pseudoknot structure (domain E) upstream of the poly(A) tail in BaMV RNA (Figure S1A), and thus domain E of the BaMV 3′ UTR might be a specifically recognized target by these proteins. To test this possibility, poly(A) and the satM10 3′ UTR, in which the BaMV pseudoknot structure is incorporated into the satBaMV 3′ UTR [29], were also used as competitors in binding assays. Findings showed that the satM10 3′ UTR had greater competition efficiency than poly(A) did (Figure 1B, lanes 11–13 and 17–19), suggesting that the three host proteins interact with the BaMV pseudoknot more efficiently than with the poly(A) tail.

To identify p87, we sliced a protein band corresponding to the binding signal from Coomassie blue-stained gel, and analyzed the sample by MALDI-TOF MS spectrometry. Mis-Fit and Mascot software yielded 10 major protein hits, 9 of which matched to Hsp90. Thereafter, p87 was identified as one of the Hsp90 family proteins of *N. benthamiana* and thus designated as NbHsp90. There are three publicly available full-length coding sequences of known *N. benthamiana* Hsp90 family proteins, namely NbHsp90-1, -2, and -3, which are reported as AY368904, AY368905, and GQ845021, respectively [46], [47]. Among the three *N. benthamiana* Hsp90 proteins, the NbHsp90 identified in this study shared the highest amino acid sequence similarity with NbHsp90-2, with an E-value of 1.28×10^−6^ and a total of 23 NbHsp90 derived peptides as identified by MALDI-TOF MS, and covering 28% of the full-length NbHsp90-2 (Figure S2, indicated by black lines). Subsequently, we designed primers based on the NbHsp90-2 nucleotide sequence and then amplified and cloned the full-length NbHsp90 coding region into pGEX-4T-1 to express NbHsp90 in *E. coli* as a GST-fusion protein, designated GST-Hsp90. The full-length amino acid sequence of NbHsp90 was determined and aligned with those of NbHsp90-1, -2, and -3 using CLUSTALW [48] (Figure S2). The sequence identities between NbHsp90 and NbHsp90-1, -2, and -3 were 96%, 99%, and 99%, respectively, indicating that there is a high conservation of amino acid sequences among the NbHsp90 isoforms. Thus, the NbHsp90 protein cloned in this study appears to be a new member of the *N. benthamiana* family of Hsp90 proteins with features shared among other previously known members.

### Characterization of direct interaction and binding domains in NbHsp90 and the BaMV 3′ UTR

**Figure 2:**
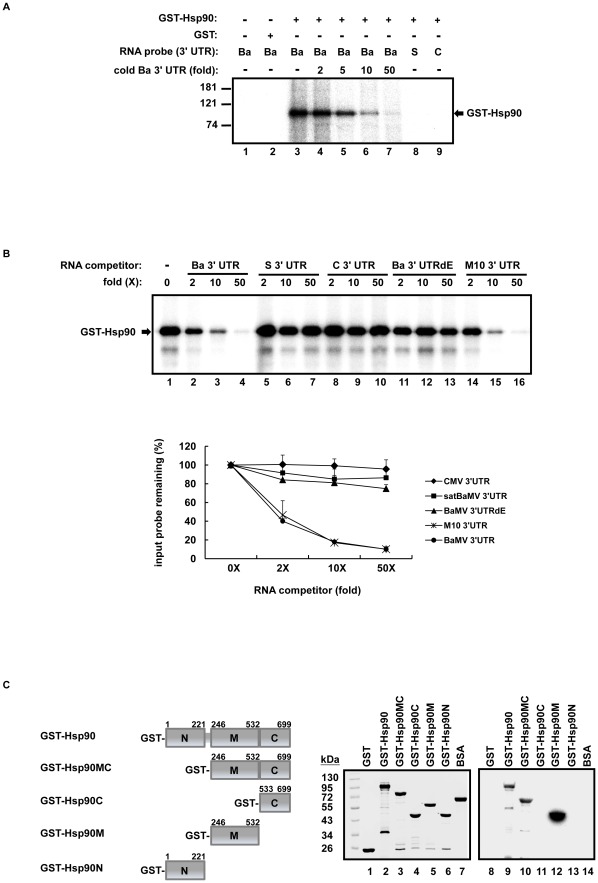
Direct interaction between NbHsp90 and the BaMV 3′ UTR. (**A**) UV cross-linking assay for the detection of direct interactions between bacterially expressed NbHsp90 with a GST-tag (GST-Hsp90) and various viral 3′ UTRs. GST-Hsp90 and GST alone were subjected to UV cross-linking assay with BaMV, satBaMV, and CMV 3′ UTR as indicated at the top of each lane. Interaction products of GST-Hsp90 and ^32^P-labeled BaMV 3′ UTR in the presence of 2-, 5-, 10-, and 50-fold molar excesses of cold BaMV 3′ UTR are shown in lanes 4–7. The positions of protein size markers are shown on the left of the figure. (**B**) Competitive UV cross-linking assays with various RNA competitors. Binding of GST-Hsp90 to the BaMV 3′ UTR in the absence of competitor or in the presence of 2-, 10-, or 50-fold molar excesses of unlabeled BaMV 3′ UTR, satBaMV 3′ UTR, CMV 3′ UTR, Ba 3′ UTRdE, or M10 3′ UTR competitors are shown as indicated at the head of each lane. The position of the expected signal representing the binding of BaMV 3′ UTR to GST-Hsp90 is indicated by an arrow. Quantification of band intensities corresponding to the 107 kDa binding signal is shown below. The intensity of the band in the treatment without competitor RNA is defined as 100%. All assays were performed in triplicate with mean averages plotted and SDs are shown as error bars. (**C**) Mapping of the BaMV 3′ UTR binding domain of NbHsp90 by Northwestern assay. Schematic representations of the GST-fused full length NbHsp90 (GST-Hsp90) and truncated forms are shown in the left panel. NbHsp90 is separated to three domains; the N-terminal (a.a.1–221), middle (a.a.246–532), and C-terminal (a.a.533–699) domains. Purified proteins (1 µg) were separated on 10% SDS-PAG and stained with Coomassie blue (lanes 1–7). The same set of proteins were separated on 10% SDS-PAG and transferred to nitrocellulose membrane, then hybridized with ^32^P-labeled BaMV 3′ UTR (lanes 8–14). The proteins were loaded as indicated at the head of each lane.

To test whether NbHsp90 interacts directly with the BaMV 3′ UTR, UV cross-linking assays were performed with purified GST-Hsp90 (Figure 2A). A binding signal with relative molecular weight of 107 kDa corresponding to GST-Hsp90 was observed only in samples containing both GST-Hsp90 and the BaMV 3′ UTR (Figure 2A, lanes 3–7). The GST tag alone did not interact with the BaMV 3′ UTR (Figure 2A, lane 2), confirming that Hsp90 is responsible for the binding of GST-Hsp90 to the BaMV 3′ UTR. By contrast, GST-Hsp90 did not interact with the CMV or satBaMV RNA 3′ UTRs, further demonstrating that there is a binding specificity between NbHsp90 and BaMV 3′ UTR (Figure 2A, lanes 8 and 9). The satBaMV and CMV 3′ UTRs did not outcompete the BaMV 3′ UTR for binding with GST-Hsp90 even at 50-fold molar excesses (Figure 2B, lanes 7 and 10). BaMV and satM10, whose 3′ UTRs contain a pseudoknot structure, competed effectively with BaMV 3′ UTR probe (Figure 2A, lanes 4–7; Figure 2B, lanes 2–4 and 14–16). The deletion of domain E in the BaMV 3′ UTR (Ba 3′ UTRdE) reduced the competitive activity to about 25% of that of the intact BaMV 3′ UTR (Figure 2B, lanes 11–13), further demonstrating that domain E in the BaMV 3′ UTR is responsible for NbHsp90 binding.

To further investigate the various molecular interactions here, we used Northwestern hybridization to determine which NbHsp90 domain is responsible for binding to BaMV 3′ UTR. NbHsp90 was divided into N-terminal (N), middle (M), and C-terminal (C) domains based on alignment results with yeast Hsp90, whose crystal structure was determined from a complex of an ATP-analogue with co-chaperone p23/Sba1 [49]. The three domains were analyzed for their RNA-binding activities with each domain alone or in combination (Figure 2C, left panel). As shown in Figure 2C, specific binding signals at positions consistent with those corresponding to the full-length GST-Hsp90, GST-Hsp90MC and GST-Hsp90M, all of which comprise the NbHsp90 M-domain (Figure 2C, lanes 9, 10, and 12), were detected. By contrast, Hsp90 mutants GST-Hsp90C and GST-Hsp90N, in which the M-domain is deleted, were unable to interact with the ^32^P-labelled BaMV 3′ UTR probe (Figure 2C, lanes 11 and 13), and neither were the control proteins GST and BSA (Figure 2C, lanes 8 and 14). These results demonstrate the existence of a binding specificity between NbHsp90 M-domain and BaMV 3′ UTR.

### Knocking down NbHsp90 reduces accumulation of BaMV RNA in *N. benthamiana*

**Figure 3:**
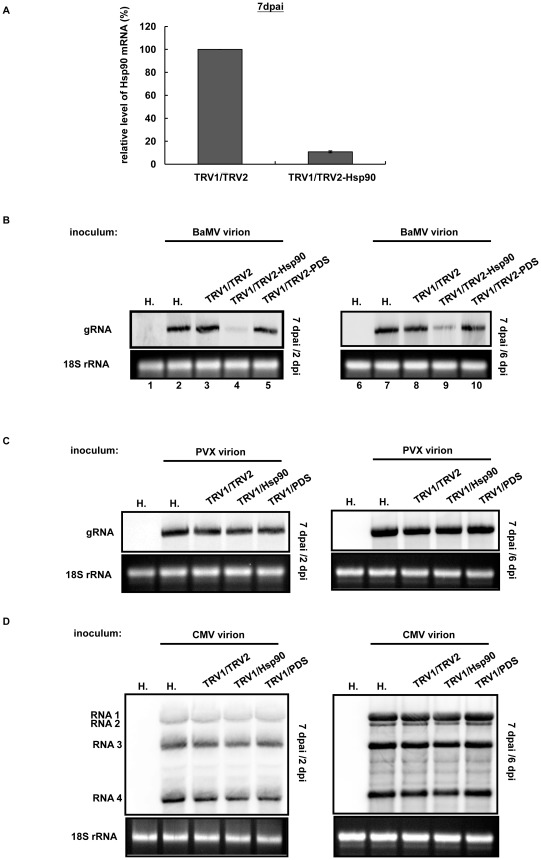
Knocking down Hsp90 expression in *N. benthamiana* reduces BaMV RNA accumulation. (**A**) Hsp90 knockdown by virus-induced gene silencing (VIGS) using the Tobacco rattle virus (TRV) vector system. Relative accumulations of NbHsp90 mRNA in control (TRV1/TRV2) or Hsp90-knockdown (TRV1/Hsp90) plants were measured at 7 dpai by real-time PCR. The Hsp90 expression in knockdown plants had reduced to about 11% of the control. The mean averages of three independent assays are shown with SDs as error bars. Frames (**B**), (**C**), and (**D**) show Northern blot analyses of the accumulation of BaMV (frame B), PVX genomic RNA (gRNA) (frame C), and CMV RNAs 1, 2, 3, and 4 (frame D) in VIGS-mediated gene-knockdown systems. Lanes designated “H.” represent healthy plants without TRV infection as negative controls. Test plants were agro-infiltrated with different constructs to induce gene silencing. At 7 dpai, BaMV, PVX, and CMV virions were used to inoculate the leaves individually. Total RNA of inoculated leaves was extracted at 2 and 6 dpi for Northern blot analysis. The ^32^P-labeled RNA probes for the detection of BaMV, PVX, and CMV RNA were complementary to the 3′-end of positive-strand RNA of the respective virus. The ethidium bromide-stained 18S rRNA is shown beneath as the loading control.

To investigate the requirement of NbHsp90 for BaMV RNA accumulation *in planta*, the TRV-based VIGS system was used to generate NbHsp90-knockdown plants. Phytoene desaturase (PDS)-knockdown plants, which exhibit the photo-bleaching phenotype 7 days post agro-infiltration (dpai), were used as indicators for the VIGS process. To examine the RNA silencing of NbHsp90, the relative level of Hsp90 mRNAs was examined at 7 dpai by real-time PCR and semi qRT-PCR (Figures 3A and S3A). It was clear that the Hsp90 mRNA level of silenced plants, TRV1/TRV2-Hsp90, was reduced to about 10% of that of the control treatment, TRV1/TRV2 (Figure 3A). To investigate the impact of Hsp90 knockdown on BaMV RNA accumulation in plants, at 7 dpai the third and fourth leaves above the infiltrated leaves were individually inoculated with BaMV, PVX, and CMV. BaMV RNA accumulation in Hsp90-knockdown plants (TRV1/TRV2-Hsp90) was reduced to 21% and 31% of levels in the control plants at 2 and 6 dpi, respectively (Figure 3B, compare lane 4 to lane 3 and lane 9 to lane 8; Figure S3B). There was no significant alteration observed in the control plants (TRV1/TRV2) or PDS-knockdown plants (TRV1/TRV2-PDS) (Figure 3B, compare lanes 3 and 5 to lane 2, and lanes 8 and 10 to lane 7) indicating that reduction in BaMV infectivity was not due to agrobacterium or TRV infection. By contrast, the accumulation of PVX and CMV RNA was not significantly affected in Hsp90-knockdown plants (Figures 3C, 3D, and S3B), revealing that the environment for virus infection in these Hsp90-knockdown plants remained unaltered. These results suggest that NbHsp90 is specifically required for the efficient accumulation of BaMV but not for PVX or an unrelated virus such as CMV.

### Hsp90 inhibitors interfere with BaMV replication in protoplasts

**Figure 4:**
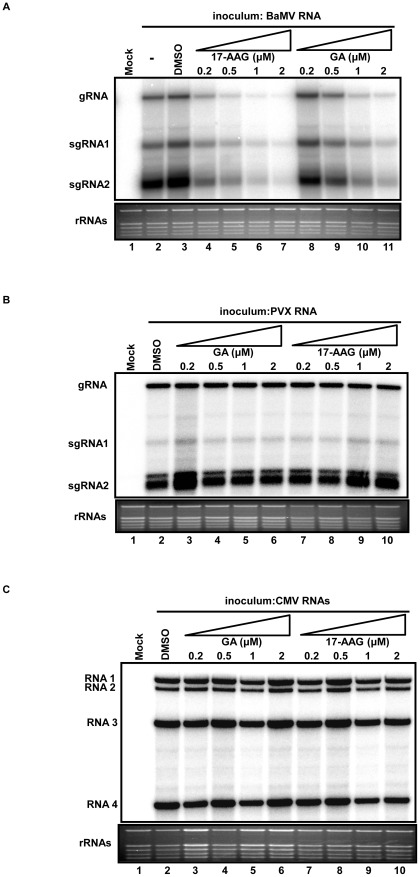
Inhibition of NbHsp90 with GA and 17-AAG treatments reduces BaMV RNA accumulation in *N. benthamiana* protoplasts. Frames (**A**), (**B**), and (**C**) show Northern blot analyses of the accumulation of BaMV, PVX, and CMV RNAs, in *N. benthamiana* protoplasts after treatment with 0.2, 0.5, 1.0, and 2.0 µM of GA and 17-AAG or the solvent DMSO. The RNA samples of protoplasts were analyzed at 24 hour post inoculation (hpi) by Northern blot hybridization. The ^32^P-labeled RNA probes for the detection of BaMV, PVX, or CMV RNA were complementary to the 3′-end of the corresponding positive strand RNAs, respectively. The ethidium bromide-stained rRNAs are shown underneath as the loading control.

The involvement of Hsp90 in the replication of animal viruses has been reported in several studies [40], [50], [51], albeit with no evidence of a direct interaction between Hsp90 and viral RNAs. To corroborate the possibility that NbHsp90 may facilitate BaMV RNA replication, we examined the effect of Hsp90 inhibitors on the accumulation of BaMV RNA. Two Hsp90-specific inhibitors, GA and the GA analogue 17-AAG, were used in this study. GA and 17-AAG bind to the Hsp90 N-terminal ATP-binding site where they block the Hsp90 chaperone function [52]. To test whether the blocked Hsp90 chaperone function interfered with the accumulation of viral RNA in protoplasts, GA or 17-AAG was added to the isolated *N. benthamiana* protoplasts for 30 min before inoculation. After incubation, 1 µg BaMV RNA was inoculated into the protoplasts and cultured in media containing 0.2 to 2 µM of GA or 17-AAG. Treatment of the protoplasts with the solvent (DMSO) alone served as a negative control and was used for normalization. The results revealed that BaMV RNA accumulation was significantly reduced for both GA and 17-AAG treatments in a dose-dependent manner, which suggests that accumulation of BaMV RNA depends on the Hsp90 function (Figures 4A and S4). DMSO alone had no effect on BaMV RNA accumulation (Figure 4A, compare lane 3 to lane 2). The inhibitory effect of 17-AAG on BaMV RNA replication was more pronounced than the inhibitory effect of GA. At a low concentration (0.2 µM), 17-AAG reduced BaMV RNA accumulation to about 30% of that in the DMSO-treated cells, whereas GA did not efficiently interfere with BaMV replication (Figure 4A, lanes 4 and 8; Figure S4). Thus, 17-AAG was used alone in subsequent experiments. To investigate the effect of NbHsp90 inhibition on the replication of other potexvirus and unrelated virus, 1 µg each of PVX and CMV RNAs was used to inoculate protoplasts individually using a similar treatment as that used for BaMV infection. The accumulation of PVX and CMV RNA was not significantly affected in GA-treated or 17-AAG-treated protoplasts (Figures 4B, 4C, and S4), which also revealed that these concentrations of Hsp90 inhibitors were not toxic to the cells. These findings are consistent with previous observations involving Hsp90-knockdown plants, again suggesting that the NbHsp90 function is specific for BaMV RNA replication.

### Hsp90 is required for efficient replication of BaMV but not for replication of satBaMV RNA

**Figure 5:**
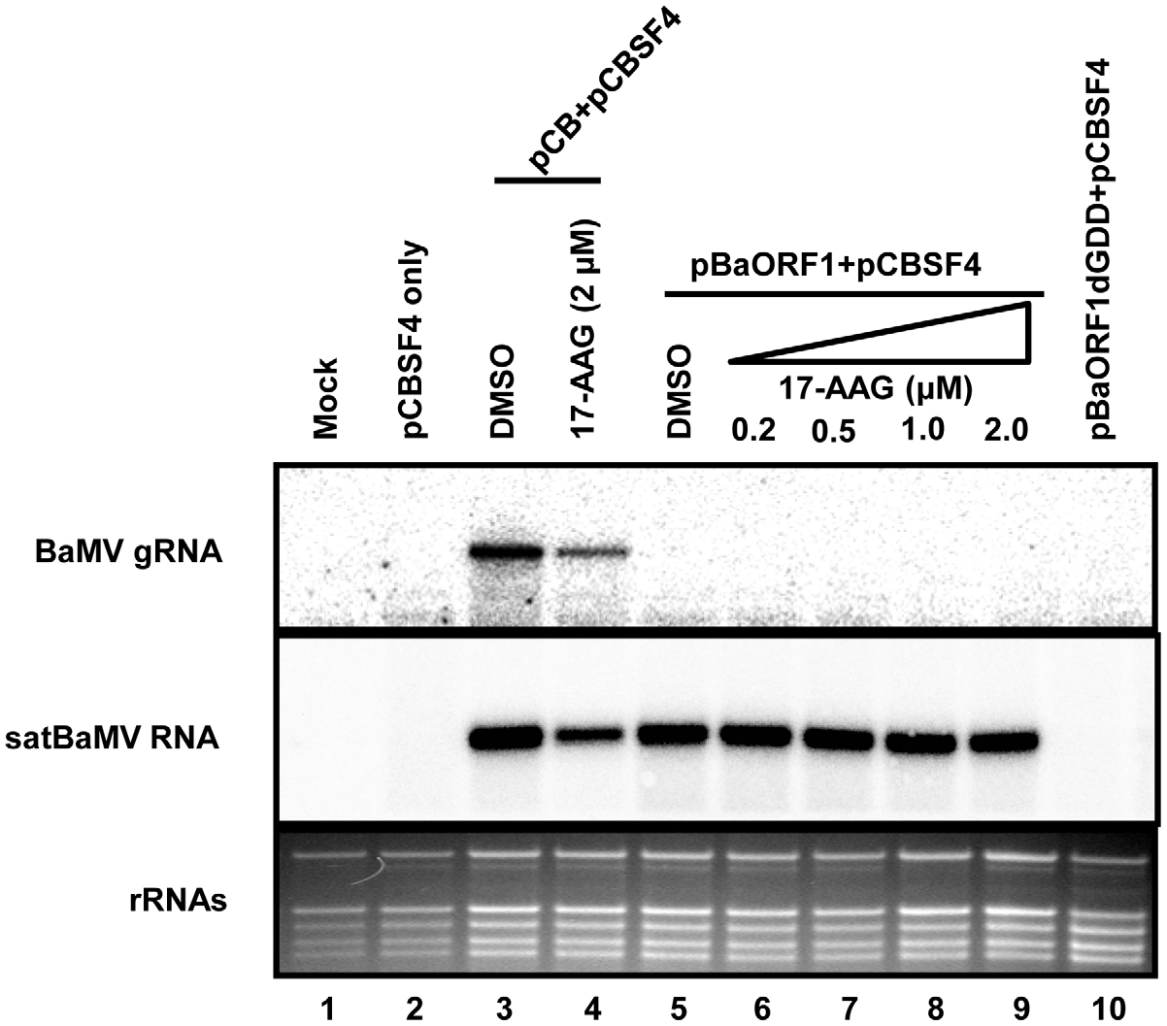
Effect of 17-AAG on satBaMV RNA accumulation in protoplasts. Northern blot analysis of total RNA prepared from inoculated *N. benthamiana* protoplasts at 24 hpi. The protoplasts were inoculated with various combinations of helper and satBaMV RNA constructs in the absence or presence of 17-AAG as indicated at the lane headings. RNA was extracted at 24 hpi, separated by electrophoresis and probed with ^32^P-labeled RNA transcripts complementary to the 3′-end of positive-stranded BaMV RNA and satBaMV RNA in the absence or presence of 17-AAG, respectively. The positions of BaMV genomic RNA (gRNA) and satBaMV RNA are indicated on the left. pCBSF4, satBaMV RNA construct; pCB, BaMV infectious clone; pBaORF1, BaMV ORF1-expressing construct; pBaORF1dGDD, defective BaMV ORF1-expressing construct, with the GDD motif of ORF1 protein deleted.

We found that NbHsp90 interacts with the BaMV 3′ UTR but not with the satBaMV 3′ UTR, implying that NbHsp90 may be required for BaMV replication but not for satBaMV RNA replication. To test this hypothesis, BaMV and satBaMV RNA infectious clones, pCB and pCBSF4 respectively, were co-inoculated into *N. benthamiana* protoplasts pretreated with 2 µM 17-AAG. We found that NbHsp90 inhibition reduces both BaMV and satBaMV RNA accumulation in protoplasts (Figure 5, lanes 3 and 4). Since satBaMV RNA depends on BaMV for replication, we thought that the reduction of satBaMV RNA accumulation might have resulted from repression of BaMV replication. To examine the requirement of Hsp90 in the accumulation of satBaMV RNA, we introduced the plasmid pBaORF1 into *N. benthamiana* protoplasts for transient expression of BaMV ORF1 protein which served as the helper for satBaMV RNA replication. As shown in lane 5 of Figure 5, the expression of ORF1 alone was able to support the replication of satBaMV RNA. By contrast, mutant pBaORF1dGDD, which encodes a nonfunctional form of ORF1 with the deletion of the GDD motif in the RdRp domain, failed to support satBaMV RNA replication *in trans* (Figure 5, lane10). These results confirmed that the native BaMV ORF1 is responsible for satBaMV RNA replication in protoplasts. The BaMV free satBaMV replication system was further adopted to analyze whether Hsp90 is required for satBaMV RNA replication. As shown in Figure 5 and Figure S5, the inhibition of Hsp90 resulting from increased concentrations of 17-AAG did not interfere with satBaMV RNA accumulation (Figure 5, lanes 5–9; Figure S5), suggesting that Hsp90 is not required for satBaMV RNA replication.

### 17-AAG inhibits BaMV RNA accumulation at the initial stage of virus infection

**Figure 6:**
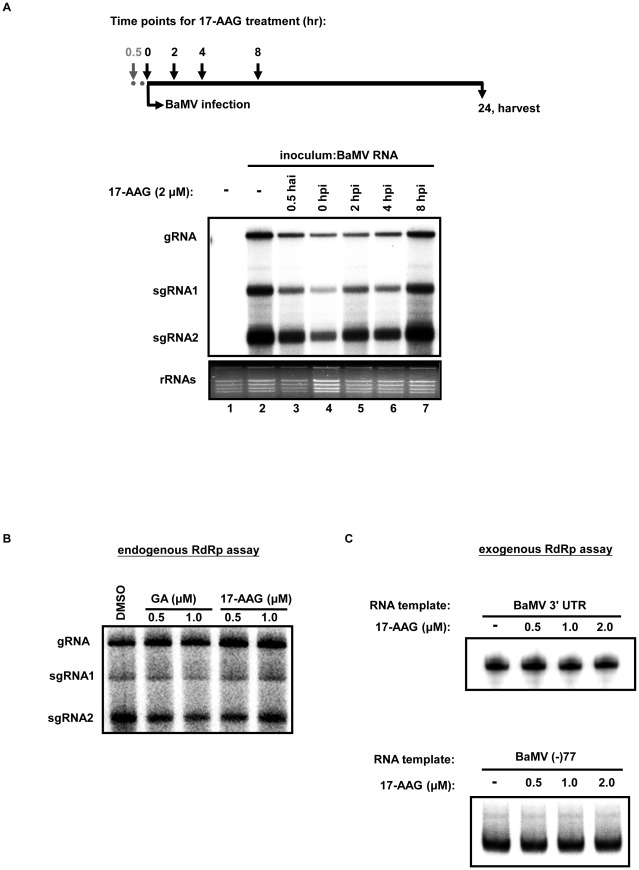
17-AAG inhibits BaMV RNA accumulation during early stage of virus infection. (**A**) Schematic representation depicting 17-AAG protoplast treatments at various time points. The protoplasts were treated with 17-AAG 0.5 hr before (indicated by the dark gray arrow and dots), or at 0, 2, 4, and 8 hr after (as indicated by numbers on top) BaMV inoculation. For treatment prior to BaMV inoculation, 17-AAG was washed out with inoculation buffer after a 30-min incubation period. The RNA samples of protoplasts were analyzed at 24 hpi by Northern blot analysis. Northern blot analyses of BaMV genomic RNA (gRNA) and two subgenomic RNAs (sgRNA 1 and 2) accumulation in protoplasts treated with 2 µM 17-AAG at various intervals before inoculation (hai — hours ante inoculation, and hpi — hours post inoculation). A “−” sign designates treatment with DMSO alone as the negative control. RNA was probed with a ^32^P-labeled RNA transcript complementary to the 3′-end of positive-stranded BaMV RNA. (**B**) and (**C**) Activities of Hsp90 inhibitor-treated BaMV RdRp preparations on endogenous and exogenous templates, respectively. The solubilized BaMV RdRp preparations were treated with various concentrations of GA or 17-AAG as indicated at the head of each lane and subjected to RdRp activity assay. Endogenous RNA (B) and exogenous RNA (C), BaMV 3′ UTR and BaMV (−)77 were used as templates for RdRp assay. The ^32^P-labeled reaction products were electrophoresed with a 1% agarose gel and analyzed by a PhosphorImager (FUJIFILM, Multi Gauge).

Hsp90 has been suggested to play an important role in the early stages of virus infection, either for efficient polymerase synthesis or for assembly of replicase complexes [36], [40]. To study the role of Hsp90 in BaMV replication in terms of different stages in the infection cycle, *N. benthamiana* protoplasts were treated with 17-AAG at various time points before or after BaMV infection. As illustrated in Figure 6A, the protoplasts were treated with 2 µM 17-AAG at 0.5 hour ante inoculation (hai), or at 0, 2, 4, and 8 hour post inoculation (hpi) of BaMV RNA and harvested at 24 hpi. For treatment at 0.5 hai, 17-AAG was washed with inoculation buffer prior to BaMV inoculation. Northern blot analysis revealed that BaMV RNA accumulation was significant reduced when 17-AAG was added before BaMV infection (0.5 hai) and during the early stages (within 4 h) after BaMV infection (Figure 6A, lanes 3–6). Protoplasts treated with 17-AAG at 8 h post BaMV infection showed no significant inhibitory effect (Figure 6A, lane 7). These observations suggest that the Hsp90 function is only required during the initial stages of BaMV replication and thus may have no impact on RNA synthesis in the later stages of replication.

17-AAG's significant inhibitory effect on BaMV RNA accumulation during the early stages of BaMV infection suggests that this inhibitory effect occurs before the maturation of BaMV replicase complexes. To test this hypothesis, a preparation of BaMV RdRp complexes purified from BaMV-infected leaves [53], [54], was used in an *in vitro* RdRp assay using both endogenous and exogenous RNA as templates. GA or 17-AAG was added to the BaMV RdRp preparations 30 minutes before the addition of [α-^32^p] UTP to initiate the endogenous RdRp assay. Newly synthesized RNA emitted radioactive signals, indicating BaMV RdRp activity. The assay showed that neither GA nor 17-AAG inhibited the synthesis of BaMV RNA *in vitro* (Figure 6B), suggesting that Hsp90 inhibition does not interfere with the RNA synthesis activity of well-assembled replicase. In the exogenous RdRp assay, endogenous RNA templates associated with the replicase complexes were removed prior to the addition of (+) or (−) BaMV 3′ terminal RNA, BaMV 3′ UTR or BaMV (−)77, as templates to assay RdRp activities on the RNA template recognition and initiation of negative- or positive-sense RNA synthesis. The results of exogenous RdRp assay revealed that 17-AAG did not interfere with both either negative- or positive-sense RNA synthesis *in vitro* (Figure 6C), even at a concentration of 2 µM. This finding supports the hypothesis that Hsp90 is involved in the initial stages of BaMV infection, and that this presumably occurs before or during the assembly process of replicase complexes.

### Yeast two-hybrid and GST pull-down assays identify an interaction between the BaMV RdRp domain and NbHsp90

**Figure 7:**
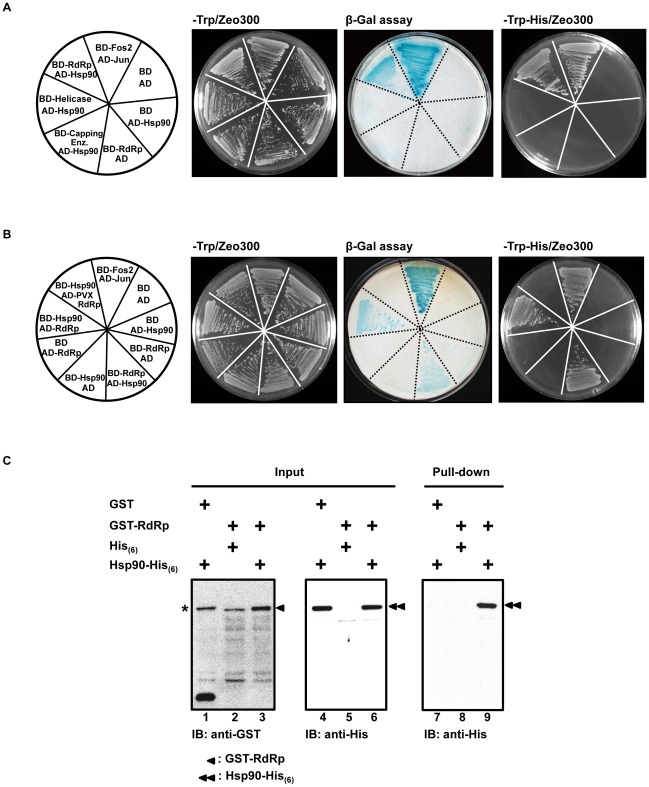
Yeast two-hybrid and GST pull-down analyses for the interaction between NbHsp90 and RdRp domain of BaMV ORF1 protein. (**A**) Mapping of NbHsp90-interacting domain of BaMV ORF1 protein by fusing each domain to Gal4-BD (BD) and NbHsp90 to Gal4-AD (AD), respectively, in the yeast two-hybrid analyses. (**B**) Reciprocal assays with exchanged fusion tags. The BaMV ORF1 protein was divided into three domains; capping enzyme, helicase, and RdRp then individually fused to BD or AD. The plasmids used to transform yeast L40 strain in each section of a media plate are as indicated on the left. Cells expressing BD-Fos2 and AD-Jun were positive controls, while those expressing BD and AD, BD and AD-Hsp90, and BD-RdRp and AD were negative controls. Colonies grown on each section of the -Trp/Zeo300 media plate result from plasmids that successfully transformed into yeast. Colonies grown on the -Trp/Zeo300 plate were transferred to filter paper for β-galactosidase activity assay. Colony formation on the selective medium (-Trp-His/Zeo300) represents the consequence of a successful interaction between the two proteins involved. (**C**) GST pull-down analysis identifies an interaction between the BaMV RdRp domain and NbHsp90. Purified *E. coli*-expressed proteins, GST, GST-RdRp, His_(6)_, and Hsp90-His_(6)_, were incubated with combinations indicated on top of each lane. Equal amount of input proteins were used (Input, left and middle panels) as demonstrated by immuno-blot assays with antibodies against GST and His-tag. Following incubation and washing, the GST affinity resin bound complexes were eluted with glutathione, resolved by electrophoresis through a 10% SDS-PAG, and analyzed by immuno-blot (IB) using His-tag antibody (as shown in the panel labeled “Pull-down”). A non-specific protein interacting with the GST antibody was indicated by the “*” sign. The positions of GST-RdRp and Hsp90-His_(6)_ were indicated by the single- and double-arrowheads, respectively.

We found that Hsp90 inhibitors GA and 17-AAG, which bind to the Hsp90 N-terminal ATP-binding site and block the chaperone function of Hsp90, interfere with BaMV replication, but do not interfere with PVX or CMV replication (Figure 4). Thus, it is conceivable that NbHsp90 might play a chaperoning role in BaMV replication. To investigate whether or not the BaMV ORF1 protein is a client protein of NbHsp90, we used a yeast two-hybrid system to analyze the interaction between BaMV ORF1 protein and NbHsp90. The three functional BaMV ORF1 protein domains—the capping enzyme domain, helicase-like domain, and RdRp core domain—were individually fused to the Gal4 DNA-binding domain (BD) to probe the interaction of NbHsp90 fusion to the Gal4 activation domain (AD). Positive interactions activate His3 expression in yeast, resulting in colony formation on plates in the absence of histidine (-Trp-His/Zeo300), and also activate LacZ, which causes an increase of β-galactosidase activity during filter assay (β-Gal assay). Plates containing zeocin in the absence of tryptophan (-Trp/Zeo300) were used to screen for successful transformation of plasmid combinations as indicated (Figures 7A and 7B). Yeast cells expressing BD-Fos2 and AD-Jun were used as positive controls. The results showed that the RdRp domain of BaMV ORF1 protein (BD-RdRp) interacts with NbHsp90 (AD-Hsp90), and that the capping enzyme domain and the helicase-like domain do not. The empty vectors (BD plus AD), BD plus AD-Hsp90, and BD-RdRp plus AD served as negative controls to exclude non-specific transactivation in reporter expression (Figure 7A). Reciprocal constructs with exchanges of BD and AD showed similar results (Figure 7B). By contrast, the RdRp domain of PVX ORF1 protein (BD-PVX RdRp) did not interact with AD-Hsp90 (Figure 7B), which is consistent with previous data showing a specific NbHsp90 inhibitory effect on the down-regulation of BaMV replication but not on PVX replication (Figure 4). The results of these yeast two-hybrid assays demonstrate that the BaMV ORF1 protein interacts with NbHsp90 through the RdRp domain. To confirm the interaction between the BaMV RdRp domain and NbHsp90, a GST pull-down assay was performed (Figure 7C). The BaMV RdRp domain and NbHsp90 were expressed and purified in *E. coli* as a GST fusion protein (GST-RdRp) and a His_(6)_ fusion protein (Hsp90-His_(6)_), respectively. GST-RdRp was bound to resin and mixed with Hsp90-His_(6)_. After incubation, GST-RdRp was eluted from the resin and Hsp90-His_(6)_ in the eluted sample was analyzed by immunoblotting. Specific signal of Hsp90-His_(6)_ was detected only when both GST-RdRp and Hsp90-His_(6)_ were present (Figure 7C, lane 9). The result further corroborated the observation that BaMV RdRp interacts with NbHsp90 *in vitro*.

## Discussion

### A new function of a host factor, NbHsp90: Specific interaction with BaMV RNA and the implication in viral RNA replication

By using the BaMV RNA 3′ UTR as a bait, we identified NbHsp90, a member of the *N. benthamiana* Hsp90 family proteins, to be a novel host factor associated with the BaMV replicase complexes. NbHsp90 is able to interact with the 3′ UTR of BaMV RNA specifically, and is required for efficient accumulation of BaMV RNA during the early stages of the infection cycle as demonstrated by the use of TRV-based VIGS and Hsp90-specific inhibitors. In contrast, NbHsp90 does not bind to satBaMV RNA or other viral RNAs, nor is it required for their replication. These findings revealed a new role of at least one member of the Hsp90 family proteins as a specific interaction partner of BaMV RNA involved in the efficient accumulation.

In terms of RNA-related functions, members of the Hsp90 family proteins have been shown to be involved in mRNA trafficking. For example, mutational studies showed that Hsp90 is important for the subcellular localization of specific mRNAs to the vicinity of mitochondria, and that the “control elements” for localization reside in the 3′ UTR of these mRNAs [55], [56]. A study on *Drosophila melanogaster* embryo development also revealed that Hsp90 is required for mRNA localization [57]. Still, there are no previous reports of a direct interaction between Hsp90 and specific mRNAs. The results of the present study represent, to our knowledge, the first direct evidence that NbHsp90 specifically interacts with a viral RNA, thus revealing a novel role for Hsp90 in viral RNA replication. The proper subcellular localization of mRNAs is important to post-transcriptional regulation of gene expression that facilitates the accurate spatial and temporal synthesis of specific proteins in cells [56]. The ability of NbHsp90 to bind specifically to the BaMV 3′ UTR raises the possibility that BaMV may have evolved to exploit the RNA-recognition function of Hsp90, and adopted this mRNA localization mechanism for the specific recruitment of BaMV genomic RNA in order to the properly assemble replicase complexes and thus initiate the replication process.

We show here that NbHsp90 directly interacts with the BaMV 3′ UTR through a pseudoknot domain (domain E) (Figure 2B). This domain is one of the key structural differences that distinguishes the BaMV 3′ UTR from the satBaMV 3′ UTR, which might contribute to NbHsp90's differential requirement for BaMV and satBaMV replication. Similarly, the PVX 3′ UTR does not fold into the pseudoknot structure (Figure S1C) [58] and does not interact with NbHsp90. This finding strongly suggests that the specific enhancement of BaMV replication by NbHsp90 is dependent on the pseudoknot structure present in the BaMV 3′ UTR. Previously, domain E of the BaMV 3′ UTR was also found to interact with viral RdRp and two host proteins, p51 and p43 [59], [60]. The addition of p51 into an RdRp reaction led to the specific down-regulation of BaMV minus-strand RNA synthesis. P43 was identified as a chloroplast phosphoglycerate kinase and as being involved in BaMV replication [60]. Thus, the BaMV 3′ UTR appears to interact with RdRp and a cohort of different host factors for replication and regulation at different stages of the infection cycle. Accordingly, like other viruses [61], the BaMV 3′ UTR may form various distinct structures that serve as ribo-switches to manage numerous events in viral replication, such as template recognition, replicase complexes assembly, and RNA synthesis. Here, we also show that the BaMV 3′ UTR interacts directly with the middle domain of NbHsp90 (Figure 2C), a conserved domain for client protein binding [32]. This suggests an alternative role for NbHsp90 in RNA chaperoning, such as the folding of the BaMV pseudoknot domain. In addition to NbHsp90's ability to bind RNA as identified in this study, other Hsps or Hsp homologues from prokaryotes to eukaryotes have also been shown to interact directly with RNA molecules [62]. For instance, the interaction between an Hsp60 homologue of the thermophilic archaeon *Sulfolobus solfataricus* and 16S rRNA is required for the cleavage of the rRNA precursor [63]. A conserved plant Hsp101 homologue directly binds to the 5′ end of TMV RNA to act as a translational enhancer [64], and an Hsp40 homologue from yeast interacts directly with nuclear tRNA or ribosomal RNA for individual regulation [65], [66]. Citing these specific interactions, the Hsps were proposed to either function directly as chaperones for mRNA molecules or to support the assembly of RNA-protein complexes in order to stabilize the RNA transcripts, or both [62]. By analogy, NbHsp90 might serve as a chaperone for the folding of the BaMV 3′ UTR structure to modulate its function in BaMV replication. This suggested role for NbHsp90 as a RNA chaperone remains to be explored.

Hsp90 has been proposed to assist in the assembly of viral replication complexes [38], [40], [41], [67] and in their proper localization [40], [41] via interaction with viral replication proteins. In this study, NbHsp90 was shown to interact with the RdRp domain of BaMV ORF1 protein in yeast cells but not with that of PVX (Figures 7A and 7B), implying that NbHsp90 specifically assists in the correct folding of BaMV ORF1 protein and in the assembly of replicase complexes through its chaperone function. This is consistent with the observation that NbHsp90 inhibition interferes with the replication of BaMV but not with that of PVX (Figure 4). Moreover, we propose that the BaMV 3′ UTR may attract NbHsp90 through a specific interaction to facilitate the proper folding and assembly of the newly translated components into functional replicase complexes.

### A model depicting the possible involvement of NbHsp90 in BaMV RNA replication

**Figure 8:**
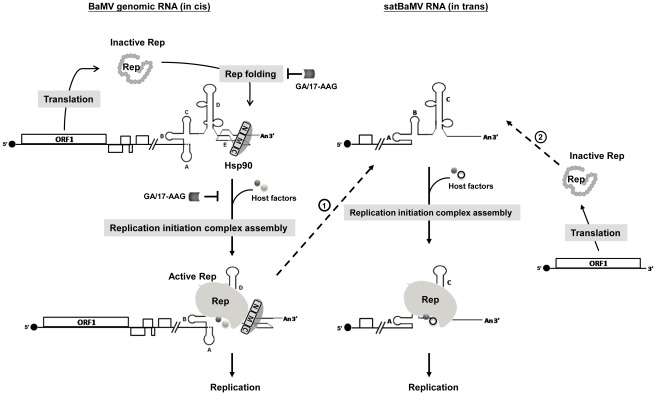
A proposed model illustrating the differential involvement of NbHsp90 in BaMV and satBaMV RNA replication. The scenario with BaMV genomic RNA is shown on the left and satBaMV RNA on the right. The 3′ UTR regions are enlarged to depict various structural domains as indicated by capital letters A through E. NbHsp90 is represented by the long oval with N-terminal (N), middle (M), and C-terminal (C) domains shown. Following infection and uncoating, ORF1-encoded protein (Rep) is translated using BaMV genomic RNA as the template. Initially, the newly translated Rep is not properly folded, as represented by the irregular string of grey circles. NbHsp90 is thought to be involved in both the proper folding of BaMV Rep and the recruitment of BaMV RNA template in the early stage of replication. Firstly, Hsp90 inhibitors GA or 17-AAG can inhibit NbHsp90, whose chaperone activity assists in the proper folding of Rep (represented by light grey ovals) and in the assembly of active replicase complexes with other host factors. Once the active replicase complexes are assembled, the GA or 17-AAG inhibitory effects (represented by the T-shaped line) are alleviated. Secondly, the NbHsp90 RNA-binding domain (M-domain), which is insensitive to inhibition by GA or 17-AAG, interacts specifically with domain E of BaMV 3′ UTR and recruits templates into the active replicase complexes. Alternatively, NbHsp90 may bind to BaMV 3′ UTR first and facilitate both the proper folding of Rep as it is newly translated and the assembly of a replication initiation complex on the BaMV 3′ UTR. By contrast, the satBaMV RNA 3′ UTR may have evolved efficient structures that serve as a scaffold for the proper folding of Rep in the absence of domain E, and facilitates the assembly of active replicase complexes with other host factors (indicated by the dashed line labeled “2”). Additionally, satBaMV RNA may utilize the preformed replicase complexes on BaMV genomic RNA for their own replication (indicated by the dashed line labeled “1”). Thus, replication of satBaMV RNA is independent of NbHsp90, and hence is insensitive to the inhibitory effects of GA and 17-AAG.

Based on results presented in this study, we propose a model (Figure 8) to illustrate the stages at which Hsp90 might participate in BaMV RNA replication. This model also highlights the differential requirement of Hsp90 for the replication of BaMV and satBaMV RNA, as well as PVX concerning the differences in structure (Figure S1). We suggest that NbHsp90 has at least two distinct functional domains involved in two distinct steps in the early stages of BaMV replication. One domain is for the protein chaperone function and is sensitive to Hsp90 inhibitors GA and 17-AAG (Figure 4). This domain is required for correct folding of the BaMV ORF1 protein and for proper assembly of replicase complexes with other host factors. Once the active replicase complexes are assembled, the GA and 17-AAG inhibitory effects are alleviated (Figure 6B). This is supported by our observation that GA and 17-AAG interference in BaMV replication decreases with increased delays in treatment after BaMV infection (Figure 6A). The other domain is required for specific recognition of domain E of the BaMV 3′ UTR, which recruits the templates into the active replicase complexes and is insensitive to GA and 17-AAG (Figure 6C). In contrast, for satBaMV RNA (Figure 8, on the right), the 3′ terminus of satBaMV RNA may have evolved effective structures that serve as a scaffold for proper folding of replicase, and to facilitate the assembly of active replicase complexes with other host factors. A similar phenomenon occurred when the BaMV ORF1 protein was expressed from the plasmid pBaORF1 (Figure 5; Figure 8, on the far right). Alternatively, satBaMV RNA may hijack the preformed replicase complexes from BaMV genomic RNA for replication. Thus, satBaMV RNA replication is independent of NbHsp90 and is therefore insensitive to the inhibitory properties of GA and 17-AAG (Figure 5).

### The differential requirement for NbHsp90 by BaMV and satBaMV RNA and the implication for helper virus specificity

SatBaMV RNA may employ replicase complexes consisting of host factor(s) distinct from those used by BaMV. The involvement of different replicases or host factors for *in cis* and *in trans* replication processes have been previously reported. For example, the genome of *Red clover necrotic mosaic virus* (RCNMV) comprises bipartite RNAs, RNA1 and RNA2. RCNMV RNA2 does not encode replicase and thus must exploit viral replicase proteins encoded by RNA1 to replicate [9]. Similarly, satRNA depends on the replicase complexes provided *in trans* by their cognate helper viruses and host plants for replication [68]. In RCNMV, the replicase component p27 is provided *in trans* and directly interacts with the RNA2 3′ UTR but not with RNA1 [69]. By contrast, only ribosome-bound RNA1 (translating template RNA) interacts with the replicase proteins, suggesting that RCNMV RNA1 is recognized in a coupling between translation and replication. This *cis*-preferential function of the virus-encoded proteins has been reported for several positive-strand RNA viruses [70]–[73]. Likewise, NbHsp90 may regulate the *cis*-preferential function of BaMV replicase by specifically binding to the BaMV pseudoknot domain, which is absent in the satBaMV 3′ UTR. Thus, BaMV may retain the replication competency in the presence of satBaMV RNA. It has been demonstrated that ribosomal RNA (rRNAs) serves as a scaffold in ribosomes for the proper positioning and folding of ribosomal proteins [74], [75]. Similarly, satBaMV RNA may have evolved distinct structural elements in the 3′ UTR that could serve as scaffolds for the proper positioning and folding of the BaMV replicase complexes in the absence of NbHsp90. Thus, the differential requirement for NbHsp90 by BaMV and satBaMV RNA might reduce the competition for replication complexes and contribute to their co-existence.

## Materials and Methods

### Viruses and satellite RNA

BaMV strain S and the associated satBaMV RNA isolate F4 [29] were used in this study. PVX, a Taiwan strain from infected potato (Liao *et al*, GenBank acc. AF272736), and CMV strain NT9 [76] were included as the controls. The purifications of BaMV, PVX, and CMV virions were carried out as described previously [77].

### BaMV RdRp preparation and RdRp assay

Methods for preparing BaMV RdRp and *in vitro* RdRp assays were described previously [54]. The preparation of exogenous templates for RdRp assay, BaMV 3′ UTR and BaMV (−)77, were described previously [54], [78]. RdRp assay products were analyzed using RNase protection assay, and quantified using a PhosphorImager (FUJIFILM, Multi Gauge).

### UV cross-linking and competition assay

UV cross-linking and competition assays were used to identify host factors directly interacting with viral RNA templates [60]. Various ^32^P-labeled RNA probes (25 fmol) in binding buffer [Tris (20 mM, pH 8.0), MgCl_2_ (3 mM), KCl (10 mM), DTT (2 mM), RNase inhibitor (5 units), yeast total RNA (1 µg), BSA (1 µg), and glycerol (4%)] were added to the BaMV RdRp preparation and incubated at room temperature for 10 min. The mixture was placed in an ice bath and illuminated under a UV lamp at 254 nm wavelength (Stratagene, UV stratalinker TM 1800) for 20 minutes. The samples were then treated with RNase A (10 µg) and RNaseT1 (0.5 unit) for 30 min at 37°C, boiled in protein sample buffer for 5 min and analyzed by electrophoresis with a 10% polyacrylamide gel containing 1% SDS. Radioactive images were scanned and quantified using the BAS-1500 bioimaging analyzer (Fujifilm, Multi Gauge). *Bam*HI-linearized pT7r138/Bam, *Xba*I-linearized psatBaMV/3′ UTR, and *Bst*NI-linearized pT7CMV/tRNA were transcribed with T7 RNA polymerase in the presence of [α-^32^P]UTP [54] for the preparation of ^32^P-labeled RNA probes corresponding to BaMV 3′ UTR, satBaMV 3′ UTR, and CMV 3′ UTR, respectively. In the competition reactions, various amounts of unlabeled competitor RNA were pre-incubated with the proteins for 10 min prior to the addition of ^32^P-labeled RNA probe. Description of competitor RNA preparations can be found in the Supporting Information (Text S1).

### Northwestern hybridization

Details for protein expression and purification are described in the Supporting Information (Text S1). Purified proteins (about 1 µg) were separated by 10% SDS-PAGE and transferred to nitrocellulose membranes. The membranes were incubated overnight at room temperature in renaturation buffer comprising Tris-HCl (10 mM), EDTA (1 mM), NaCl (50 mM), yeast total RNA (25 µg/ml), and 1× Denhardt's reagent at pH 7.5. The membranes were probed with ^32^P-labelled BaMV 3′ UTR (5×10^5^ cpm/ml) for 2 h and washed with renaturation buffer three times (10 min per wash). The membrane was then air dried and analyzed by PhosphorImager (FUJIFILM, Multi Gauge).

### VIGS and virus challenging

The *Tobacco rattle virus* (TRV)-based virus induced gene silencing (VIGS) system [79] was used for knocking down the expression of specific host genes. The pTRV1, pTRV2, and pTRV2/PDS plasmids were kindly provided by Dr. David C. Baulcombe (Department of Plant Sciences, University of Cambridge, UK). Plasmid pTRV2/NbHsp90 harboring a 300-bp fragment corresponding to nt 1801–2100 of the NbHsp90 coding sequence was constructed by PCR using pGEXHsp90 as the template with the forward primer NbHsp90S-F (5′- GCTCTAGAAGCAAGAAGACCATG-3′) and reverse primer NbHsp90S-R (5′- TCCCCCGGGTTAGTCAACTTCC-3′), followed by cloning of the amplified fragment into the pTRV2 vector. The pTRV1, pTRV2, and pTRV2/NbHsp90 plasmids were individually introduced into *Agrobacterium tumefaciens* strain C58C1 by electroporation. For VIGS assays, *A. tumefaciens* cultures (OD_600_ = 1) containing pTRV1, pTRV2, or pTRV2/NbHsp90 were mixed in a 1∶1 ratio as indicated, and co-infiltrated by syringe onto three leaves of each test plant (*N. benthamiana*). At 7 days post agro-infiltration (dpai) the third and fourth leaves above the infiltrated sites were mechanically inoculated with 0.5 µg virions of BaMV, PVX, or CMV. Two and six days later, total RNA was extracted from the virus-inoculated leaves of three independent plants. BaMV, PVX, and CMV RNA accumulations were determined by Northern blot assay.

### Real-time PCR

At seven dpai, total RNA of the third and fourth leaves above the infiltrated leaves were extracted for detection of Hsp90 mRNA expression by real-time PCR. First-strand cDNA was synthesized by reverse transcription using 1 µg of total RNA as the template and oligo d(T)_39_ primer. For real-time PCR, the Hsp90 mRNA levels were monitored by forward primer Hsp90-908F (5′-AGGGTCAGCTGGAGTTCAA-3′), and reverse primer Hsp90-1098R (5′-GGGAAGATCCTCGGAATCCAC-3′). For a negative control, we used PCR without prior reverse transcription. The actin mRNA level was determined by real-time PCR with forward primer Actin-F (5′-GATGAAGATACTCACAGAAAGA-3′) and reverse primer Actin-R (5′-GTGGTTTCATGAATGCCAGCA-3′) for normalization of the specifically silenced gene.

### RNA analysis by Northern blot

Total RNA was extracted from inoculated leaves at 2 and 6 dpi and from protoplasts at 24 hpi [77]. RNA samples were separated by electrophoresis after denaturation in the presence of glyoxal and transferred onto nylon membrane (Amersham, UK) for Northern blot analysis [77]. Blots were hybridized with specific riboprobes to detect BaMV, PVX, CMV, or satBaMV RNA. The ^32^P-labeled probes, specific for the detection of (+)-strand BaMV and satBaMV, were transcribed from *Hind*III-linearized pBaHB and *Eco*RI-linearized pBSHE using SP6 and T7 RNA polymerase, respectively, as described previously [80]. The PVX- and CMV-specific probes were prepared by linearization of pPVXHE and pCMVHE harboring PVX and CMV (−) 3′ terminal sequences downstream of the T7 promoter, respectively (Hsu *et al*, unpublished), with *Hind*III, followed by transcription with T7 RNA polymerase in the presence of [α-^32^P] UTP. Hybridization signals were detected and quantified by using a PhosphorImager (Multi Gauge, FUJIFILM).

### Protoplast isolation, Hsp90 inhibitor treatment, and viral RNA infection

Protoplasts were isolated from *N. benthamiana* as previously described [27]. Hsp90 inhibitors, geldanamycin (GA, Stressgen) and 17-allylamino-demethoxygeldanamycin (17-AAG, Sigma-Aldrich) were used at 0.2 to 2 µM concentration for the inhibition of ATP-Hsp90 interaction in the protoplast cells. Varying concentrations of GA or 17-AAG were added to the isolated protoplasts before or after virus infection at the indicated times. For each inoculation, 1 µg of viral RNA or 10 µg of plasmid DNA of infectious cDNA clone was used to inoculate 2×10^5^ protoplasts. BaMV, PVX, or CMV RNA was purified as described previously [77]. The infectious clones of BaMV-S and satBaMV RNA, pCB and pCBSF4, respectively, were described previously [29], [80]. To express BaMV ORF1 and its derived mutant in protoplast cells, plasmids pBaORF1 and pBaORF1dGDD, a defective ORF1 with the GDD motif deleted [26], were generated from the full-length pCB and pCBdGDD clones respectively, by removing other ORFs using *Dra*III and *Sac*I.

### Yeast two-hybrid assay

Yeast strain L40, pHybLex/Zeo bait and pYESTrp2 prey vectors, and positive control plasmids pHybLex/Zeo-Fos2 and pYESTrp-Jun were from the Hybrid Hunter Kit purchased from Invitrogen. In a two-hybrid system, we divided the transcription factor into two domains, a DNA binding domain (BD) and an activation domain (AD). These were fused as two separated hybrid proteins referred to as bait and prey, respectively. A positive interaction causes *HIS3* and *lacZ* reporter gene expression in *Saccharomyces cerevisiae*. Preparation of yeast competent cell, yeast transformation, selection of interaction, and β-galactosidase filter assay were carried out according to the user manual provided by Invitrogen. The bait and prey designate plasmids combined as indicated were transformed simultaneously into an L40 cell and plated on -Trp/Zeo300 for selection of successfully co-transformed cells. The colonies grown on the selected plate were transferred to the filter to analyze β-galactosidase activity. Additionally, three colonies were selected at random, dissolved in water and plated on -Trp-His/Zeo300 for interaction selection. Descriptions of plasmids and their construction are detailed in the Supporting Information (Text S1).

### GST pull-down assay

Details for protein expression and purification are described in the Supporting Information (Text S1). GST or GST fusion proteins (1 µg each) were mixed with GST affinity resin (Novagen) and shaken gently at 4°C for 1 h, followed by the addition of His_(6)_ fusion proteins (1 µg each) and further incubation with gentle shaking for 2 h. The proteins bound to beads were washed with 1× PBS and eluted with elution buffer (50 mM Tris-HCl, pH 8.0, 10 mM glutathione). The eluted proteins were resolved by 10% SDS-PAGE and analyzed by immunoblotting using an anti-His antibody (LTK BioLaboratories).

## Supporting Information

Figure S1 **Schematic representation of the secondary structures of the 3′ UTRs of BaMV, satBaMV, and PVX.** (**A**) The secondary structure of the BaMV 3**′** UTR, established by enzymatic and chemical probing, comprises four stem-loops (domains A, B, C, and D) and a pseudoknot (Domain E) [27]. (**B**) The secondary structure of the satBaMV 3**′** UTR, confirmed by enzymatic probing, comprises stem-loops A, B and C [29]. (**C**) The secondary structure of the PVX 3**′** UTR, established by enzymatic and chemical probing, consists of stem-loops 1, 2 and 3 [58].
(TIF)

Figure S2 **Comparison of the NbHsp90 amino acid sequences with those of three known isoforms.** The full-length amino acid sequences of all published Hsp90 isoforms, NbHsp90-1, NbHsp90-2, and NbHsp90-3, and that of the clone from our lab, NbHsp90, were aligned using CLUSTAL W. The alignment output is ordered by the similarity between NbHsp90 and NbHsp90-1, -2, and -3. NbHsp90 is most similar to NbHsp90-2. Residues are numbered below the alignment. Completely conserved residues from the 4 sequences are lettered in gray. Residues that are identical to NbHsp90 are lettered in black and similar residues are lettered in white over a gray background. Different residues are lettered in white over a black background. The black lines above the sequence show the peptides identified by MALDI-TOF MS.
(TIF)

Figure S3 **Knocking down Hsp90 expression in** ***N. benthamiana*** **reduces BaMV RNA accumulation.** (**A**) Semi-quantitative RT-PCR analysis of Hsp90 mRNA levels in silenced (TRV1/TRV2-Hsp90) and non-silenced (TRV1/TRV2, Mock, and TRV1/TRV2-PDS) plants. Actin mRNA was used as an internal control. Lane NC represents the negative controls in which the reverse transcriptase-free RT reaction mix was used as a template in the PCR reaction (36 cycles). (**B**) Quantification of the relative accumulation of BaMV, PVX, and CMV viral RNA in the control and NbHsp90-silenced plants at 2 or 6 dpi. All data are mean averages of three experiments and normalized against those of TRV1/TRV2 control plants.
(TIF)

Figure S4 **Inhibition of NbHsp90 with GA and 17-AAG treatments reduces BaMV RNA accumulation in** ***N. benthamiana*** **protoplasts.** Quantification of relative accumulation of BaMV, PVX, and CMV viral RNAs in control and Hsp90 inhibitor-treated protoplasts was shown. The data are mean averages of three independent experiments and normalized against those of DMSO-treated control protoplasts.
(TIF)

Figure S5 **Effect of 17-AAG on satBaMV RNA accumulation in protoplasts.** The relative accumulation of satBaMV RNA in protoplasts treated with DMSO or 17-AAG at various concentrations as indicated at the bottom. The satBaMV RNA accumulation concentration in DMSO treated protoplasts is designated as 100%. The data represent the mean averages of three independent experiments and standard deviations are indicated.
(TIF)

Text S1 **Details for plasmid construction and recombinant protein purification.**
(DOC)
