# Supplementary figures and images for: Hsp90 Interacts Specifically with Viral RNA and Differentially Regulates Replication Initiation of Bamboo mosaic virus and Associated Satellite RNA
Source: PLoS Pathog. 2012 May 24;8(5):e1002726. doi: 10.1371/journal.ppat.1002726 (PMC3359997; doi:10.1371/journal.ppat.1002726)

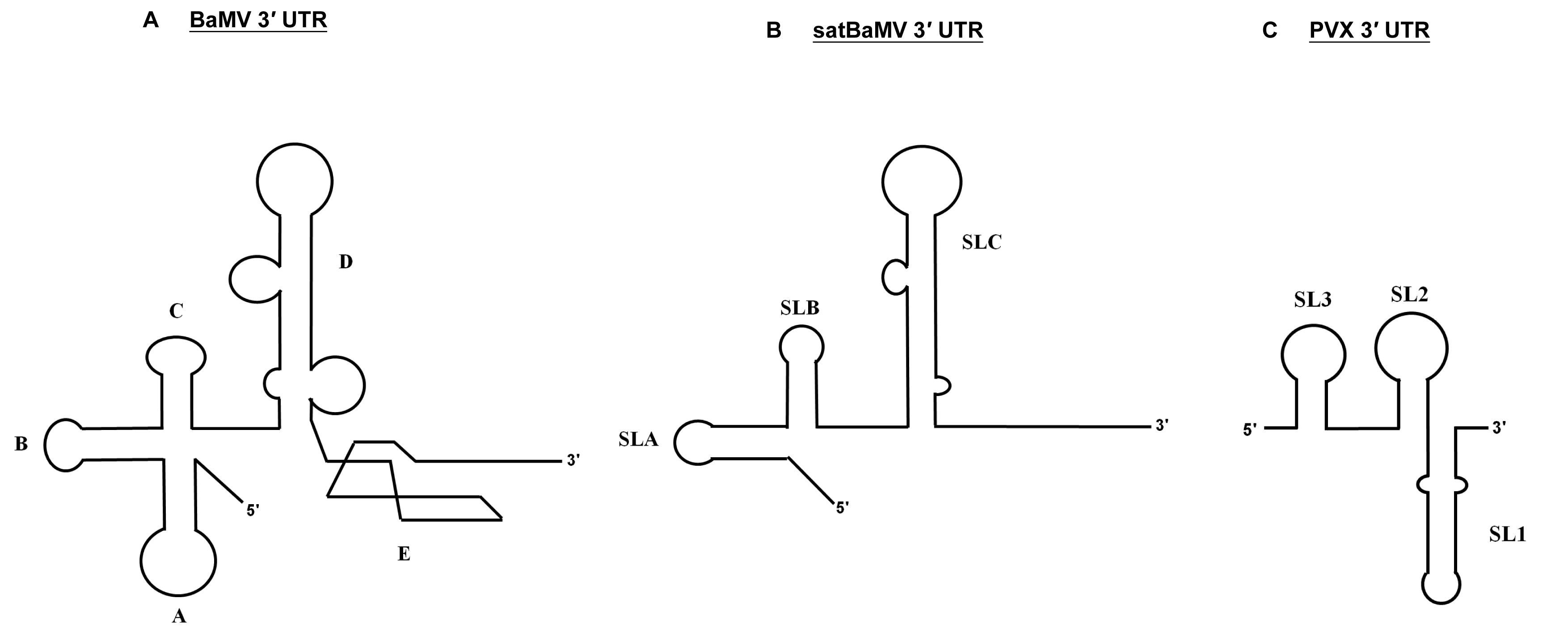

Supplement: Figure S1 — Schematic representation of the secondary structures of the 3′ UTRs of BaMV, satBaMV, and PVX. (A) The secondary structure of the BaMV 3′ UTR, established by enzymatic and chemical probing, comprises four stem-loops (domains A, B, C, and D) and a pseudoknot (Domain E) [27]. (B) The secondary structure of the satBaMV 3′ UTR, confirmed by enzymatic probing, comprises stem-loops A, B and C [29]. (C) The secondary structure of the PVX 3′ UTR, established by enzymatic and chemical probing, consists of stem-loops 1, 2 and 3 [58]. (TIF) [file ppat.1002726.s001.tif]

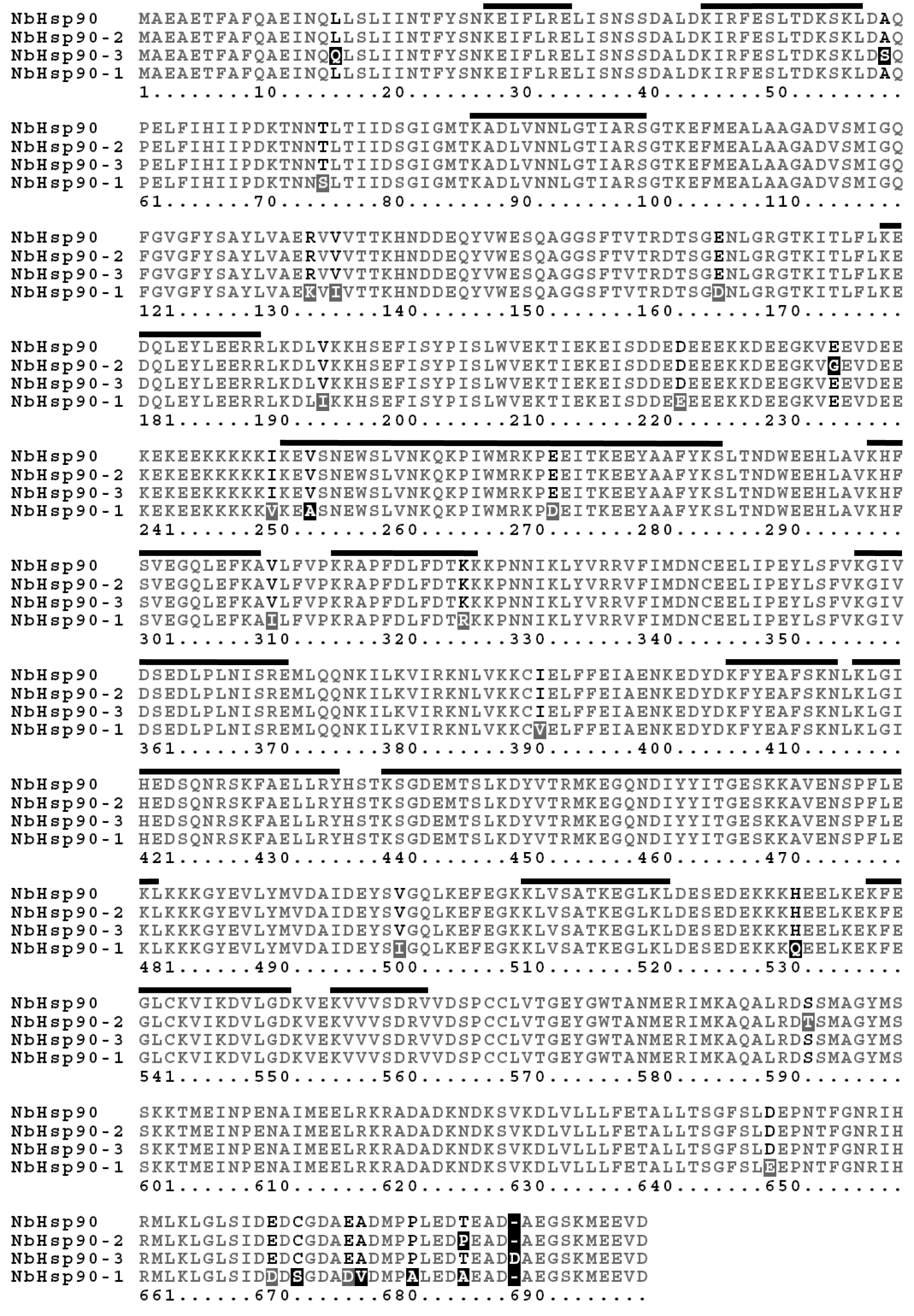

Supplement: Figure S2 — Comparison of the NbHsp90 amino acid sequences with those of three known isoforms. The full-length amino acid sequences of all published Hsp90 isoforms, NbHsp90-1, NbHsp90-2, and NbHsp90-3, and that of the clone from our lab, NbHsp90, were aligned using CLUSTAL W. The alignment output is ordered by the similarity between NbHsp90 and NbHsp90-1, -2, and -3. NbHsp90 is most similar to NbHsp90-2. Residues are numbered below the alignment. Completely conserved residues from the 4 sequences are lettered in gray. Residues that are identical to NbHsp90 are lettered in black and similar residues are lettered in white over a gray background. Different residues are lettered in white over a black background. The black lines above the sequence show the peptides identified by MALDI-TOF MS. (TIF) [file ppat.1002726.s002.tif]

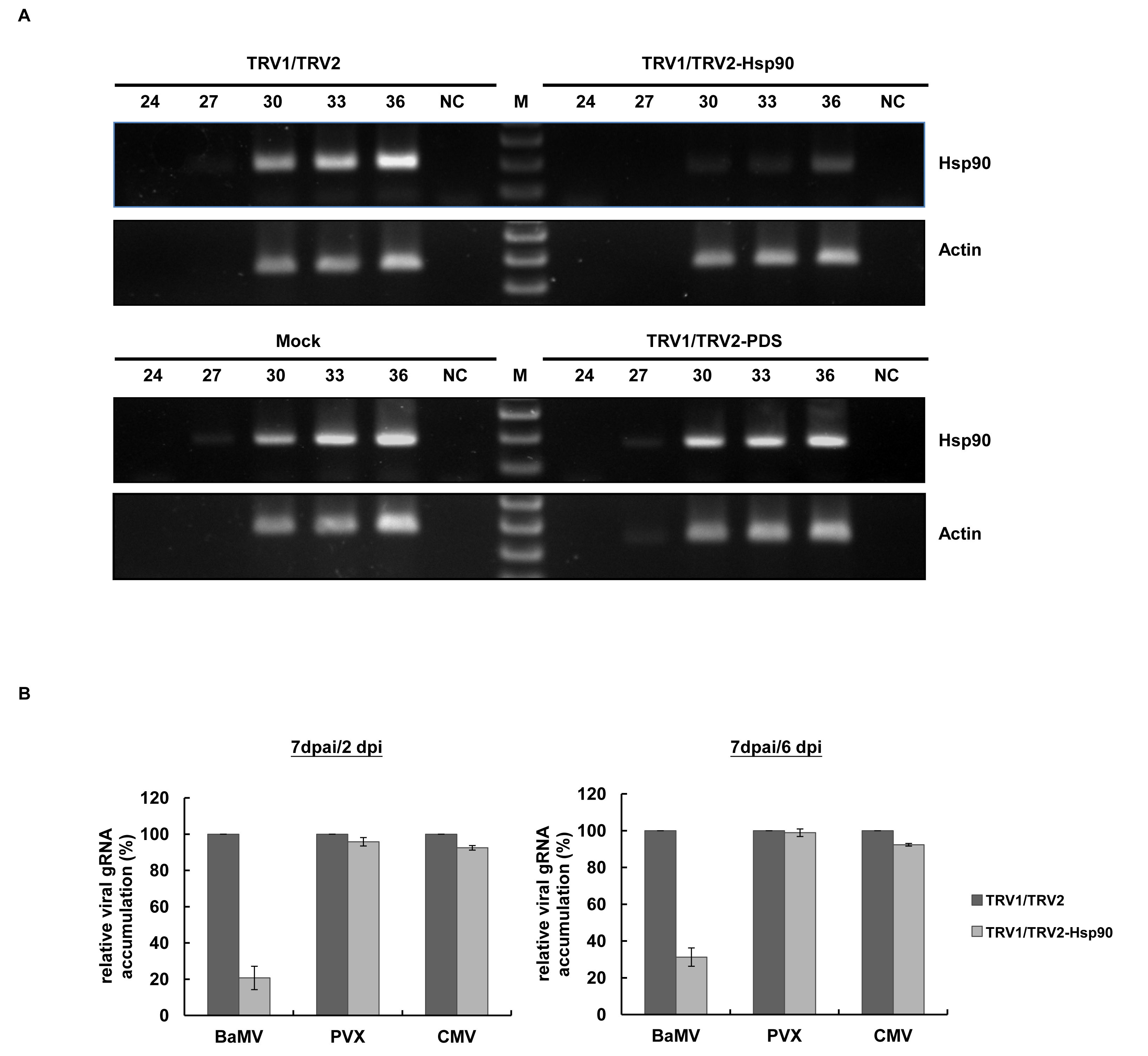

Supplement: Figure S3 — Knocking down Hsp90 expression in N. benthamiana reduces BaMV RNA accumulation. (A) Semi-quantitative RT-PCR analysis of Hsp90 mRNA levels in silenced (TRV1/TRV2-Hsp90) and non-silenced (TRV1/TRV2, Mock, and TRV1/TRV2-PDS) plants. Actin mRNA was used as an internal control. Lane NC represents the negative controls in which the reverse transcriptase-free RT reaction mix was used as a template in the PCR reaction (36 cycles). (B) Quantification of the relative accumulation of BaMV, PVX, and CMV viral RNA in the control and NbHsp90-silenced plants at 2 or 6 dpi. All data are mean averages of three experiments and normalized against those of TRV1/TRV2 control plants. (TIF) [file ppat.1002726.s003.tif]

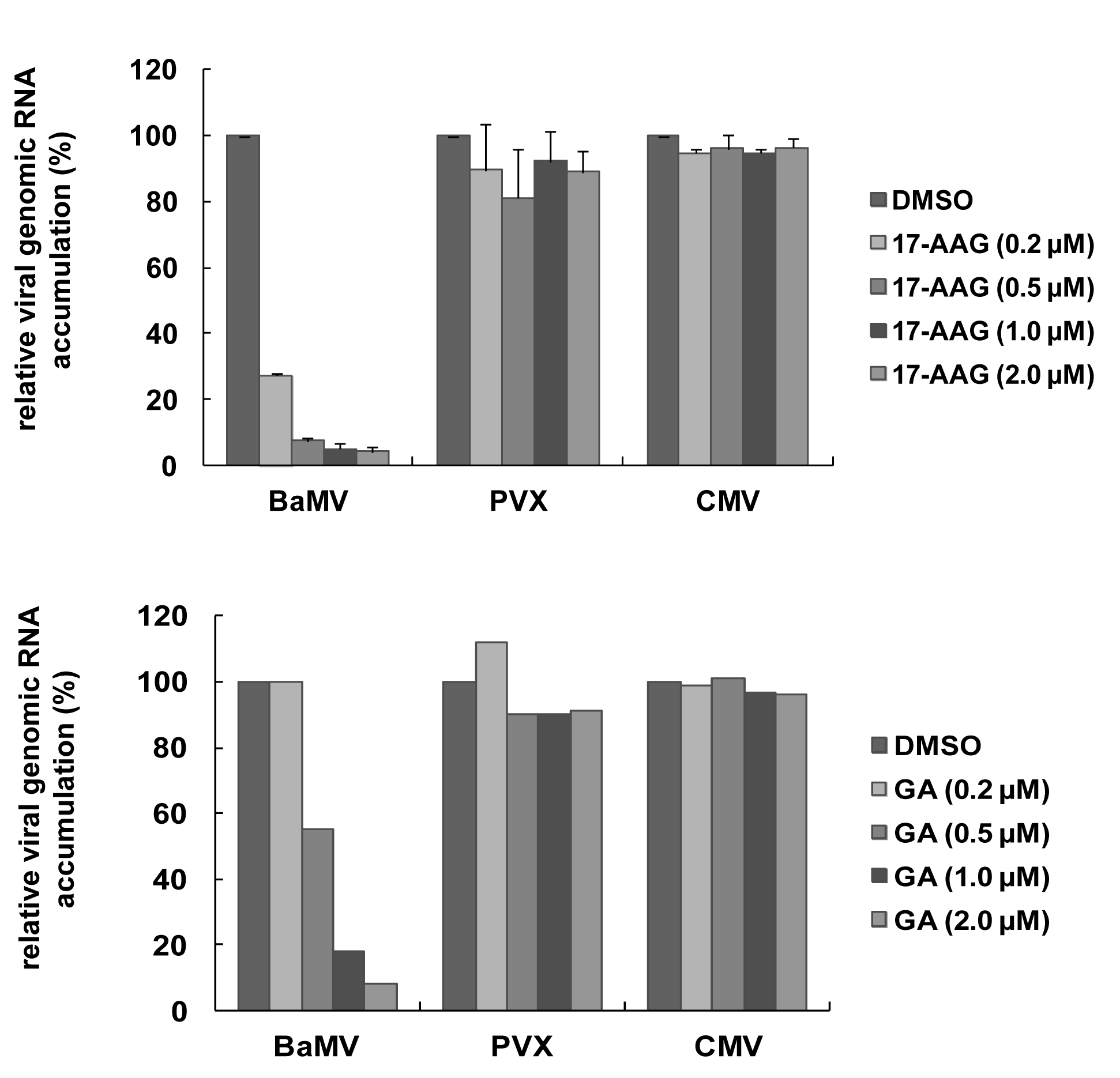

Supplement: Figure S4 — Inhibition of NbHsp90 with GA and 17-AAG treatments reduces BaMV RNA accumulation in N. benthamiana protoplasts. Quantification of relative accumulation of BaMV, PVX, and CMV viral RNAs in control and Hsp90 inhibitor-treated protoplasts was shown. The data are mean averages of three independent experiments and normalized against those of DMSO-treated control protoplasts. (TIF) [file ppat.1002726.s004.tif]

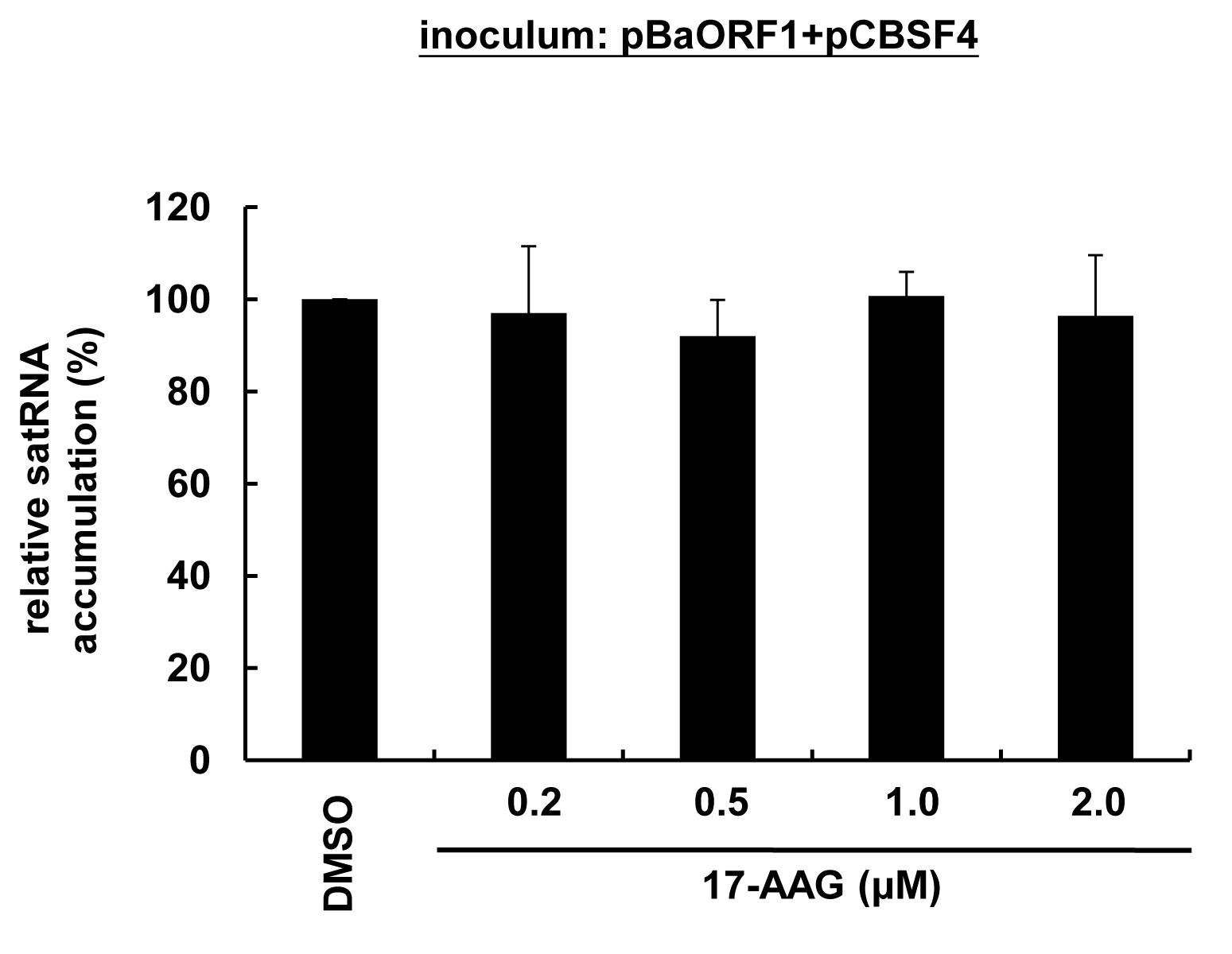

Supplement: Figure S5 — Effect of 17-AAG on satBaMV RNA accumulation in protoplasts. The relative accumulation of satBaMV RNA in protoplasts treated with DMSO or 17-AAG at various concentrations as indicated at the bottom. The satBaMV RNA accumulation concentration in DMSO treated protoplasts is designated as 100%. The data represent the mean averages of three independent experiments and standard deviations are indicated. (TIF) [file ppat.1002726.s005.tif]
