# Supplementary material for: Hsp90 Interacts Specifically with Viral RNA and Differentially Regulates Replication Initiation of Bamboo mosaic virus and Associated Satellite RNA
Source: PLoS Pathog. 2012 May 24;8(5):e1002726. doi: 10.1371/journal.ppat.1002726 (PMC3359997; doi:10.1371/journal.ppat.1002726)
Supplement: Text S1 — Details for plasmid construction and recombinant protein purification. (DOC) [file ppat.1002726.s006.doc]

**Text S1. Details for plasmid construction and recombinant protein purification.**

**Preparation of BaMV and satBaMV RNA 3′ UTR mutants as competitors**

To analyze the elements responsible for host factor interactions, BaMV and satBaMV RNA 3′ UTR mutants were prepared and used as competitors in assays. The plasmid psatM10/3′ UTR, harboring the 3′ UTR region of the M10 mutant of satBaMV RNA, was constructed by PCR amplification from pBSM10 [1] with 5′ primer EcoRIT7BS710 (5′-CCGGAATTCTAATACGACTCACTATAGGATCCACGAGCACAACCG-3′) and 3′ primer BS-M10 [1]. The BaMV 3′ UTR domain E deletion plasmid, pBa/3′ UTRdE, was PCR amplified from pCB [1] as the template with 5′ primer T7/Ba3′+138 [2], and 3′ primer, Ba(-)6342 (5′-CTAGTCTAGAGGTCTTTATTCTGCTG-3′). All of the PCR products were gel purified, phosphorylated and individually cloned into the *Sma*I digested and dephosphorylated vector pUC119. RNA competitors were prepared by in vitro transcription with the *Xba*I-linearized plasmids psatM10/3′ UTR, pBa/3′ UTRdE individually as the template with T7 RNA polymerase. RNA homopolymers, poly(A) and poly(IC), were obtained from Sigma-Aldrich, Inc.

**Expression and purification of recombinant proteins**

We constructed the pGEXHsp90 plasmid to express GST-fused Hsp90 (GST-Hsp90) in *E. coli*. A cDNA fragment corresponding to the NbHsp90 open reading frame was amplified from the primers SalI/Hsp90F (5′-CACGCGTCGACTCATGGCGGAGGCAGAGAC-3′) and NotI/Hsp90R (5′-ATAAGAATGCGGCCGCTTAGTCAACTTCCTCCATCTTGCTAC-3′) by reverse transcription-PCR using *N. benthamiana* total RNAs as templates. The gel-purified PCR products were digested with *Not*I and *Sal*I and ligated to pGEX-4T-1. Of the ten sequenced clones, six harbored the same Hsp90 coding sequence and these six were selected for recombinant protein production. To determine the NbHsp90 domain responsible for BaMV 3′ UTR binding, four deletion mutants, pGEXHsp90MC, pGEXHsp90C, pGEXHsp90M, and pGEXHsp90N were constructed. Plasmids pGEXHsp90MC and pGEXHsp90C were constructed by PCR amplification from pGEXHsp90 as template, with 3′primer Not I/Hsp90R and 5′ primers SalI/Hsp90-736F (5′-CACGCGTCGACTCAAGAAAAAGAAGAAG-3′) and XhoI/Hsp90-1597F (5′-CCGCTCGAGGAAGAACTAAAGGAG-3′), respectively. The Hsp90M DNA fragment was amplified by PCR using pGEXHsp90 as template with 5′ primer SalI/Hsp90-736F, and 3′ primer NotI/Hsp90-1596R (5′- ATAAGAATGCGGCCGCTTAGTGCTTTTTCTTCTCATC-3′). The Hsp90N DNA fragment was amplified by PCR using pGEXHsp90 as template with 5′ primer SalI/Hsp90F and 3′ primer NotI/Hsp90-663R (5′-ATAAGAATGCGGCCGCTTAATCATCAGAGATTTC-3′). PCR products were gel purified, digested with appropriate restriction enzymes and cloned into the pGEX-4T-1vector.

To produce the recombinant GST, GST-Hsp90 and deletion forms, a 0.5-ml overnight culture of *E. coli* BL21 (DE3) cells harboring the appropriate plasmid was transferred into 500 ml of medium and incubated at 37°C to mid-log phase. Isopropyl-β-D-thiogalactopyranoside (IPTG) (1 mM final concentration) was added at 30°C to induce protein expression. The cells were harvested by centrifugation after 10 h incubation. The pellets were resuspended in sonication buffer [50 ml, 1× PBS, 10 mM DTT, and protease inhibitor cocktail (Roche Diagnostics)] and sonicated with a sonifier (Sonicator 3000, Misonix) at 4°C with 15% amplitude at 10-sec intervals for 20 min. The cell extract was clarified by centrifugation at 12,000 rpm for 10 min then mixed with GST affinity resin (1 ml) (Novagen) and shaken gently at 4°C for 1 h. The resin mixture was then washed with buffer (3 × 5 ml, 1× PBS) and eluted with elution buffer (1 ml, 50 mM Tris-HCl, pH 8.0, 10 mM glutathione) to obtain the partially purified recombinant proteins. The partially purified proteins were dialyzed in DEAE binding buffer (20 mM Tris-HCl, pH 8.0, 20 mM NaCl, 10% glycerol) and subsequently passed through a DEAE-column (1 ml column volume) (Amersham Pharmacia Biotech) at a flow rate of 1 ml/min. The bound GST-Hsp90 and its deletion forms, were eluted with 20–1000 mM NaCl gradient (12 ml).

**Plasmid construction for yeast two-hybrid assay**

The plasmids for expressing the BD and AD fused proteins in yeast were generated with pHybLex/Zeo and pYESTrp2 vectors respectively. Plasmids were constructed by PCR amplification from pCB templates with the primer pairs EcoRI/cap-F (5′-GCGGAATTCATGGCACTCGTTTC-3′) and PstI/cap-R (5′-AAAACTGCAGTTAGGCATCTTTTG-3′) for pLexcap, EcoRI/helicase-F (5′-GCGGAATTCATTGACGTGCTCG-3′) and PstI/helicase-R (5′-AAAACTGCAGTTAAGAGTGTTCCTTTG-3′) for pLexhelicase, and EcoRI/RdRp-F (5′-GCGGAATTCCTGCCTGACCCGCAG-3′) and PstI/RdRp-R (5′-AAAACTGCAGTTAACTAGAG-3′) for pLexRdRp. The pLexHsp90 plasmid was constructed by PCR amplification using pGEXHsp90 as template, with the primer pair: ApaI/Hsp90-F (5′-TTGGGCCCGATGGCGGAGGCAGAG-3′) and Not I/Hsp90R. PCR products were gel purified, digested with the appropriate restriction enzymes and cloned into pHybLex/Zeo vectors. For construction of pYESHsp90 and pYESRdRp, the DNA fragments of Hsp90 and BaMV RdRp were digested, respectively, from pGEXHsp90 and pGEXRdRp with *Bam*HI and *Not*I and then ligated to pYESTrp2. The pYESPVXRdRp plasmid was generated by PCR amplification using pPVX-T, a cDNA infectious clone of PVX (Liao et al, GenBank accession number AF272736), as template, from the primer pair BamHI/PVXRdRp-F (5′-CGGGATCCAAGGAGTACGAGCC-3′) and NotI/PVXRdRp-R (5′-ATAAGAATGCGGCCGCTTAAAGAAAGTTTCTG-3′). PCR products were gel purified, digested with appropriate restriction enzymes and cloned into pYESTrp2 vectors.

**Plasmid construction and protein purification for GST pull-down assay**

For expressing the BaMV RdRp as a GST fusion protein (GST-RdRp), the plasmid pGEXRdRp was constructed by PCR amplification using pCB as the template and the primer pairs, BamHI/Ba2773F (5′-CGGGATCCCTGCCTGACCCGCAG-3′) and SalI/Ba4191R (5′-ACGCGTCGACTTAACTAGAGAATAAAC-3′). The gel-purified PCR products were digested with *Bam*HI and *Sal*I and ligated to pGEX-4T-1 (GE Healthcare Life Science). The plasmid pETHsp90, for expressing NbHsp90 as a His_(6)_ fusion protein (Hsp90-His_(6)_), was constructed by PCR amplification using pGEXHsp90 as the template with the primer pairs, NcoI/Hsp90F (5′-CATGCCATGGCGGAGGCAG-3′) and NotI/Hsp90R-NS (5′-ATAAGAATGCGGCCGCGTCAACTTCCTCCATCTTGCTAC-3′). The gel-purified PCR products were digested with *Nco*I and *Not*I and ligated to pET29a (Novagen). The expression and purification of GST-RdRp were the same as described above for those of GST-Hsp90. For purification of Hsp90-His_(6)_, the culture of the recombinant *E. coli* BL21 harboring pETHsp90 was treated with 1 mM IPTG for 10 h at 30°C. The cells were disrupted by sonication and the cell extract was clarified by centrifugation at 12,000 rpm for 10 min. The supernatant was subjected to a DEAE-column (Amersham) and the target protein was subsequently purified through a HisTrap HP Column (Amersham).

**References**

1. Huang YW, Hu CC, Lin CA, Liu YP, Tsai CH, et al. (2009) Structural and functional analyses of the 3' untranslated region of Bamboo mosaic virus satellite RNA. Virology 386: 139-153.

2. Cheng JH, Ding MP, Hsu YH, Tsai CH (2001) The partial purified RNA-dependent RNA polymerases from bamboo mosaic potexvirus and potato virus X infected plants containing the template-dependent activities. Virus Res 80: 41-52.
